# Speed accuracy trade-off under response deadlines

**DOI:** 10.3389/fnins.2014.00248

**Published:** 2014-08-15

**Authors:** Hakan Karşılar, Patrick Simen, Samantha Papadakis, Fuat Balcı

**Affiliations:** ^1^Department of Psychology, Koç UniversityIstanbul, Turkey; ^2^Department of Neuroscience, Oberlin CollegeOberlin, OH, USA

**Keywords:** response deadlines, optimality, speed-accuracy, timing uncertainty, decision making

## Abstract

Perceptual decision making has been successfully modeled as a process of evidence accumulation up to a threshold. In order to maximize the rewards earned for correct responses in tasks with response deadlines, participants should collapse decision thresholds dynamically during each trial so that a decision is reached before the deadline. This strategy ensures on-time responding, though at the cost of reduced accuracy, since slower decisions are based on lower thresholds and less net evidence later in a trial (compared to a constant threshold). Frazier and Yu (2008) showed that the normative rate of threshold reduction depends on deadline delays and on participants' uncertainty about these delays. Participants should start collapsing decision thresholds earlier when making decisions under shorter deadlines (for a given level of timing uncertainty) or when timing uncertainty is higher (for a given deadline). We tested these predictions using human participants in a random dot motion discrimination task. Each participant was tested in free-response, short deadline (800 ms), and long deadline conditions (1000 ms). Contrary to optimal-performance predictions, the resulting empirical function relating accuracy to response time (RT) in deadline conditions did not decline to chance level near the deadline; nor did the slight decline we typically observed relate to measures of endogenous timing uncertainty. Further, although this function did decline slightly with increasing RT, the decline was explainable by the best-fitting parameterization of Ratcliff's diffusion model (Ratcliff, 1978), whose parameters are constant within trials. Our findings suggest that at the very least, typical decision durations are too short for participants to adapt decision parameters within trials.

## Introduction

**Figure 1:**
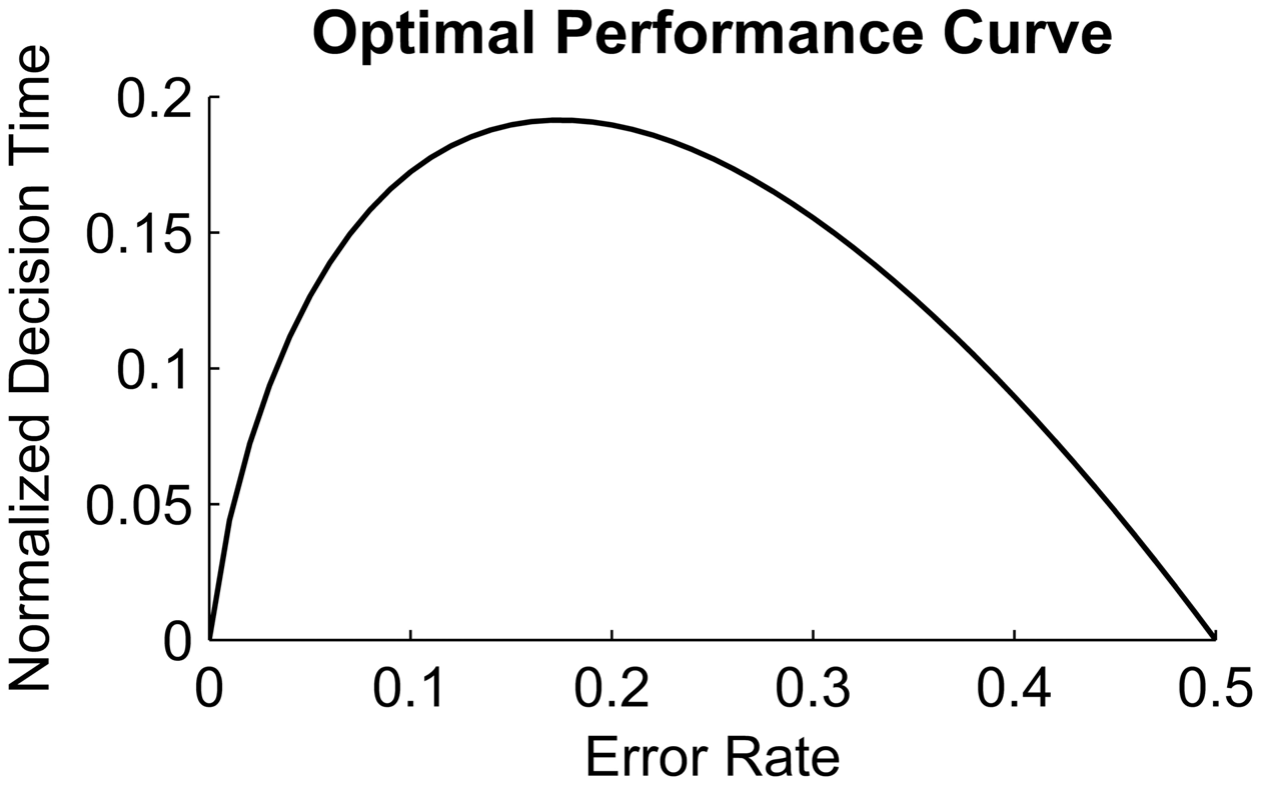
**Optimal Performance Curve derived from the pure Drift Diffusion Model**. Horizontal axis shows ER and vertical axis shows the normalized decision time, i.e., Decision time divided by RSI + Ter; Reproduced from Bogacz et al. (2006).

Noisy evidence accumulation models such as the drift-diffusion model (DDM, Ratcliff, 1978, 1981, 1985, 1988, 2002) have successfully explained accuracy and RT patterns in two-alternative forced choice (2AFC) perceptual decision tasks. The DDM has also been useful in defining an optimality-based benchmark for decision making. For instance, Bogacz et al. (2006) formulated a parameter-free optimal performance curve (OPC; Figure 1) relating the DDM's decision speed to its accuracy in a class of 2AFC tasks. Specifically, on tasks in which the signal-to-noise ratio (SNR) stays constant within a test block and within trials, the two stimulus types are equally likely and participants are free to wait as long as they wish prior to responding. The OPC prescribes an optimal normalized decision time (DT) for a given level of accuracy in order to maximize the expected reward rate (RR) in such free-response paradigms. If the signal quality is very high, then little evidence needs to be accumulated to achieve high accuracy; conversely if there is no signal in the environment (necessarily yielding an error rate around 0.5), the decision maker should accumulate little or no evidence before making a choice. In this way, the participant can maximize the number of decisions made (trials generated) in a fixed amount of test duration. However, when the SNR is at an intermediate level, the optimal decision strategy requires accumulating more evidence (and thus generating fewer trials) for maximizing the RR; the maximum decision time is associated with accuracy levels of roughly 0.8. Note that the OPC for 2AFC tasks was defined based on the assumptions of the reduced DDM analyzed by Bogacz et al. (2006), which lacks the between-trial variability of the core parameters found in Ratcliff's DDM.

**Figure 2:**
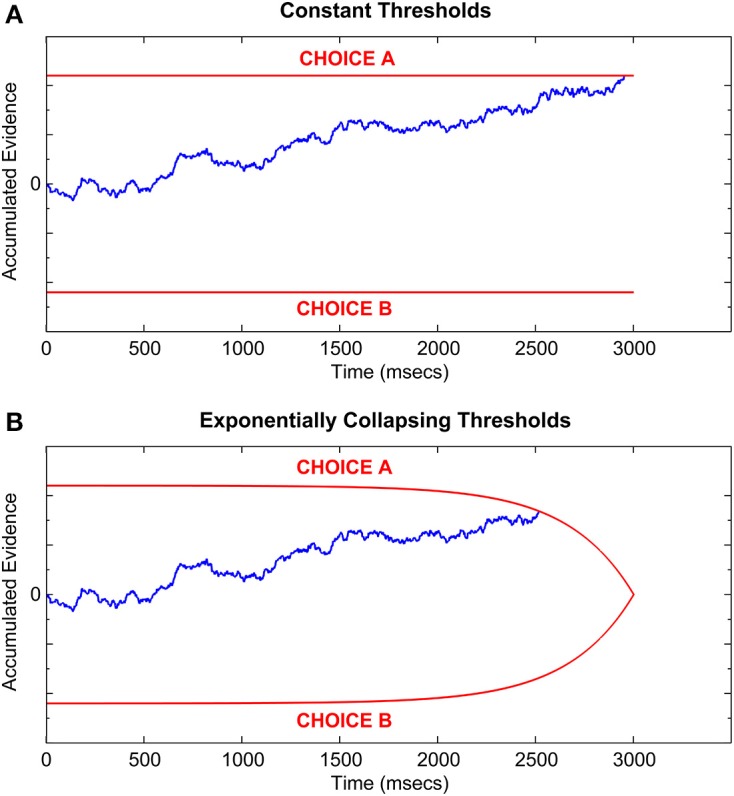
**Sample Drift-Diffusion process with (A) constant thresholds, and (B) exponentially collapsing thresholds which meet at a hypothetical deadline of 3000 ms**. Red lines represent the two decision thresholds; blue line represents the evidence accumulation process [identical in both **(A,B)**]. Threshold crossing time represents the decision time. Total RT equals decision time plus non-decision latency.

Inherent in the formulation of the OPC is a trade-off between speed and accuracy of decisions (SAT; Wickelgren, 1977), which posits that fast responses suffer from less evidence accumulation and are thus less accurate, whereas slower responses benefit from more evidence accumulation resulting in higher accuracy at the cost of time. In formal decision making models such as the DDM, SAT is represented by a threshold parameter that determines how much evidence is accumulated in favor of each hypothesis in a 2AFC task (Figure 2). A higher threshold requires more evidence accumulation and thus underlies a slower response, on average, whereas a lower threshold leads to a faster response at the expense of an increased chance of errors due to noisy evidence accumulation (e.g., Ratcliff and McKoon, 2008). Research shows that, with extensive training, participants can maximize their RR by setting the optimal threshold, which defines the optimal trade-off between the speed and accuracy of their decisions (e.g., Simen et al., 2009; Balcı et al., 2011b). However, behavioral studies testing for optimality in 2AFC paradigms typically do not enforce hard time constraints on the decision making process (e.g., Feng et al., 2009; Simen et al., 2009; Bogacz et al., 2010; Starns and Ratcliff, 2010, 2012; Balcı et al., 2011b), which provides a theoretically infinite (in reality limited by the test block duration) amount of time to the participant before a decision must be made.

Decisions in real life scenarios rarely enjoy such temporal luxury for gathering evidence, but instead often need to be terminated before a pre-specified deadline, after which no reward can be earned (e.g., in class exams). Optimal behavior in such settings requires the decision maker to collapse decision thresholds as the deadline approaches, such that they meet when the deadline is reached, in order to secure at least a 50% chance of earning a reward, as opposed to a 0% chance if responding late. In this regard, see Frazier and Yu (2008), who analyzed optimal threshold collapse for a loss function that linearly combines an indicator of on-time, accurate responding, the RT itself, and a penalty for late responding. This loss function is closely related, but not identical, to an objective function equaling the RR. As such, the notion of time-dependent collapsing thresholds (or similarly, time-dependent inflation of evidence accumulation rates) has received a great deal of attention in the decision making literature (Luce, 1986; Rao, 2010; Drugowitsch et al., 2012; Thura et al., 2012).

Two interesting hypotheses emerge from this formulation. First, a higher level of endogenous timing uncertainty (for a fixed deadline) requires an earlier threshold collapse, along with a lower rate of decline (see Frazier and Yu, 2008; Figures 2A,B). Within this formulation, endogenous timing uncertainty refers to the trial-to-trial variability in a participant's estimates of time intervals (Buhusi and Meck, 2005). Second, for a given level of timing uncertainty, threshold collapsing should begin earlier for a shorter deadline. Balcı et al. (2011a) tested these previously untested predictions in a pilot study but found little evidence of collapsing thresholds; however, their design might not have been optimized to investigate these predictions that might have obscured signs of threshold collapse (e.g., not terminating the RDM stimulus at the deadline). This study tests these predictions more rigorously, and thereby elucidates the extent to which optimal behavior in 2AFC is achievable when reward maximization entails within-trial modulation of decision thresholds. Additionally, we aim to investigate the extent to which, if at all, participants are successful in factoring their level of timing uncertainties into their threshold modulation.

In order to formally define the optimal 2AFC behavior, whether under response deadlines or not, we need mathematical models which can accurately describe accuracy along with RT in 2AFC tasks by relying on various psychomechanistic components underlying a complete decision making process. One such model is the above-mentioned DDM, which conceptualizes decision making as a bounded, noisy, evidence accumulation process (Figure 2) in the form of an ongoing computation of the current log-likelihood ratio of the two hypotheses under consideration (Stone, 1960). At its core, the DDM is a continuous version of the Sequential Probability Ratio Test (SPRT), which is a statistical procedure for minimizing the number of samples necessary to decide between two hypotheses with a given mean accuracy, as well as maximizing the likelihood of arriving at the correct hypothesis for any given number of samples (Wald and Wolfowitz, 1948). In the formulation of the DDM, the step time between the samples accumulated in an SPRT becomes infinitesimal, resulting in a continuous random walk, where the duration from the start of the evidence accumulation until a threshold crossing represents the decision time (see Stone, 1960).

The drift-diffusion process is defined by the stochastic differential equation:

(1) dx=Adt+cdW

Here, as in Bogacz et al. (2006), *x* denotes the difference between the evidence supporting two different alternatives at time *t*, *Adt* represents the average increase in *x* during the interval *dt*, and *cdW* is Gaussian distributed white noise with mean 0 and variance *c*^2^*dt* (Ratcliff and Smith, 2004). When *x* crosses one of the two decision thresholds (one above the starting point, and one below it) a decision is made. This threshold crossing time represents the decision time. Within this formulation the drift rate *A* represents the average rate of the evidence accumulation, and is the slope of this random walk process. On the other hand, the noise component explains the random fluctuations in the same process and accounts for the fact that a given SNR can lead to correct decisions in some trials and errors in some others. This model is now referred to as the pure DDM (Figure 2; see Bogacz et al., 2006). It uses RT and accuracy data in order to describe decision performance by quantifying drift rate (*v*; rate of evidence accumulation), boundary separation (*a*; decision threshold), non-decision related latency (*T*_*er*_), and starting point (*z*) parameters. In a more generalized version, three parameters of the DDM (*v, z*, and *T_er_*) were made variable on a trial-by-trial basis, mainly to allow for fitting data with unequal average RT for correct and incorrect responses (Ratcliff and Rouder, 1998) and is appropriately named the extended DDM (see Bogacz et al., 2006).

The DDM has been successful in explaining RT and accuracy data in various psychophysical studies (see Voss et al., 2013 for a review) including recognition memory (Ratcliff, 1978; McKoon and Ratcliff, 2012), brightness discrimination (Ratcliff, 2002), color discrimination (Spaniol et al., 2011), and even the classification of clinical disorders (Mulder et al., 2010; White et al., 2010). Of greater relevance to this study, however, is the DDM's utilization in prescribing unique threshold parameters for RR-maximizing (i.e., optimal) performance in 2AFC tasks. As mentioned earlier, the theoretical work by Bogacz et al. (2006) has defined a closed-form RR-maximizing function that prescribes a specific average decision time for each error rate (ER), and also defines the OPC. Bogacz et al. (2010) and Simen et al. (2009) have tested the extent to which human participants are optimal in setting RR-maximizing thresholds, and have found that within a single session, thresholds were generally set too high compared to their optimal values. Balcı et al. (2011b) have replicated this finding, but have also shown that this accuracy bias diminishes with practice.

Bogacz et al. (2010) and Balcı et al. (2011b) argued that sub-optimal performance due to favoring accuracy over reward rate (observed in their studies after a limited level of training) might be an adaptive threshold setting bias that takes into account endogenous timing uncertainty. This adaptive bias was attributed to the asymmetry (i.e., lower rate of decline in RR for thresholds higher than the optimal threshold) in the RR curves as a function of decision threshold (Bogacz et al., 2006; Figure 15), which entails that setting the threshold higher than the optimal threshold leads to a higher RR than setting it too low by the same amount. A more adaptive response under endogenous timing uncertainty therefore entails favoring slower yet more accurate responses (Bogacz et al., 2006; Balcı et al., 2011b). Balcı et al's (2011a) findings suggest that participants can “monitor” their levels of uncertainty regarding temporal properties of the task, and thereby factor it into the decision process. This proposition is further supported by studies showing that humans and other animals can in fact take normative account of their timing uncertainties at both sub- and supra-second intervals in order to reach optimal performance when they make decisions based on the durations of stimuli/events (e.g., Hudson et al., 2008; Balcı et al., 2009; Jazayeri and Shadlen, 2010; Simen et al., 2011; Çavdaroğlu et al., 2014; for a review see Balcı et al., 2011a). Overall, these studies suggest that timing uncertainty is instrumental in shaping choice behavior and determining how much reward is earned both in temporal and non-temporal decision-making. The importance of interval timing to perceptual decision making is further emphasized by recent studies proposing possible mechanisms (e.g., gain modulation) by which temporal information processing can modulate speed-accuracy tradeoffs (e.g., Standage et al., 2011, 2013).

Endogenous timing uncertainty becomes even more relevant to optimal choice behavior in 2AFC perceptual decision making when a response deadline is explicitly introduced to the decision process. Such situations are familiar to most organisms in their natural settings, within which contextual temporal properties constantly require arriving at a decision before a stochastic deadline. For instance, correctly identifying when and how long a prey will be available in a hunting ground, as well as which prey to hunt among the alternatives (“Slow but old?,” “Young but fast?”) are of vital importance for a predator's survival. The optimal predator in its attempt to choose the best option should also require less and less information for arriving at a decision as the time for the prey animals to leave approaches. This strategy ensures that it catches at least one prey, though perhaps not an ideal one, instead of losing all. Moreover, it should engage in this decision process while simultaneously relying on its level of uncertainty regarding how much time it has before a choice must be made. If it is too uncertain about temporal intervals, or the time until the prey animals leave is too short, the predator should start reducing the required level of evidence earlier, and should at worst pick a random prey right before the time to leave, if it still hasn't done so. This hypothetical naturalistic scenario exemplifies the above-mentioned optimal strategy in a situation with a response deadline, which is to collapse the decision threshold such that by the time the deadline is reached, a response of at least 50% accuracy is ensured.

Two main hypotheses emerge under this scenario. First, for a given deadline, higher timing uncertainty makes it necessary to collapse thresholds earlier compared to lower timing uncertainty, so that the deadline is not passed by accident, ultimately resulting in an opportunity cost. Second, for a given timing uncertainty, participants need to start collapsing decision thresholds earlier for shorter deadlines, compared to longer ones. Frazier and Yu (2008) have shown that both predictions should manifest themselves with steady decline in accuracy as time approaches the deadline, which should closely parallel the presumed decline in decision thresholds. We can quantify this time-dependent decline in thresholds by calculating accuracy levels for RTs bins of a specific size. The resulting curve formed by connecting the accuracy levels in these bins constitutes the conditional accuracy (a.k.a. Micro Speed Accuracy Trade-off) curve (Wickelgren, 1977; Luce, 1986). Since the diffusion process calculates the log-likelihood ratio of the two hypotheses, a particular accuracy level is assured by setting a particular decision threshold. When accuracy data is sorted and binned in this way, this principle should still hold for each individual RT bin. Thus, if the threshold is dynamically set lower in later time bins, then by definition this also prescribes lower accuracy for those bins (Luce, 1986).

**Figure 3:**
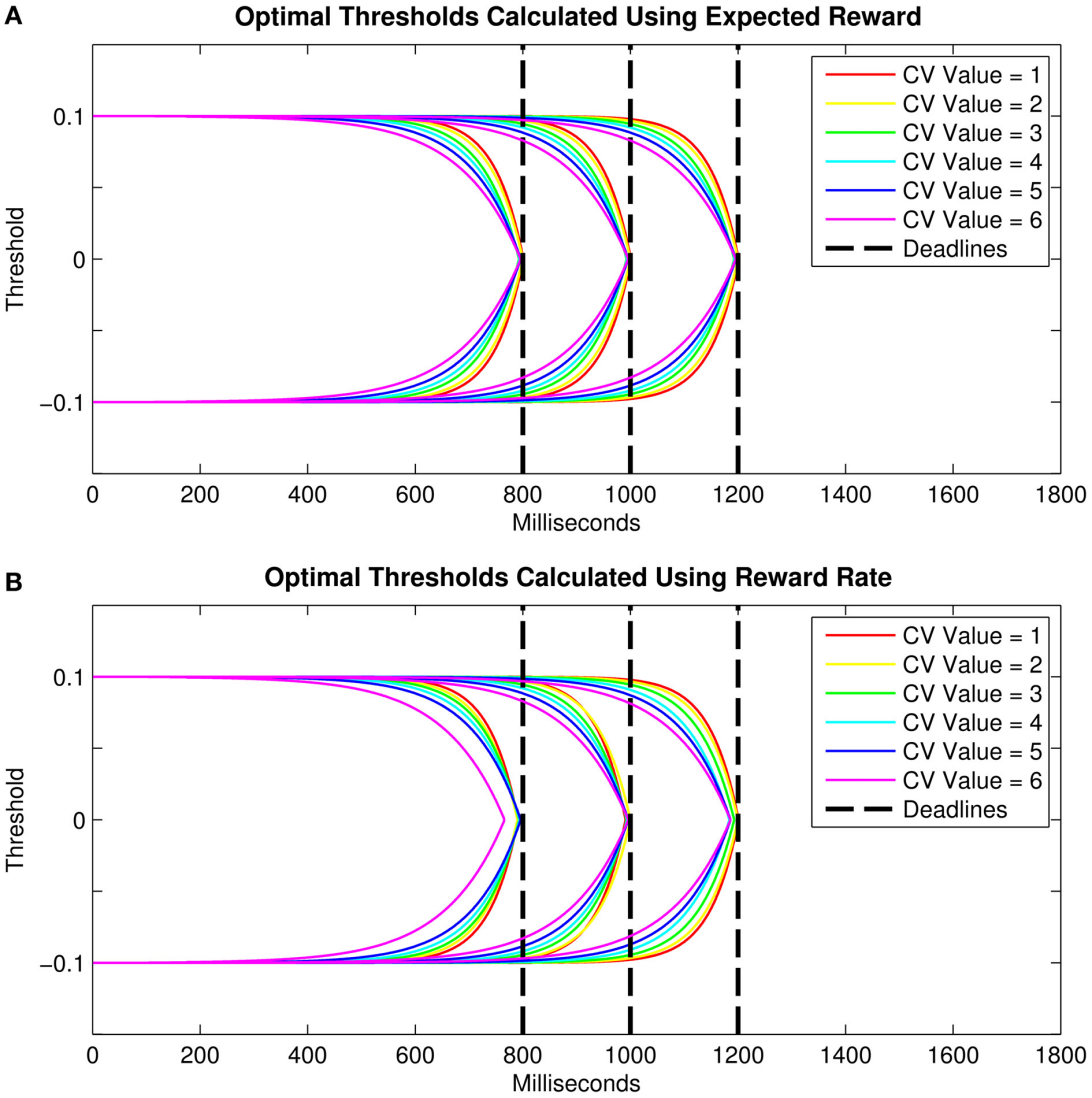
**Optimal threshold collapse trajectories selected from the family of exponential decline functions for three different response deadlines and six hypothetical levels of timing uncertainty when optimality criterion is taken as (A) the expected total reward, and (B) reward rate**. Vertical dashed lines represent the response deadlines (800, 1000, and 1200 ms).

Here, we conduct simulations in order to approximate the optimal relationship between threshold collapsing and (1) the deadline duration and (2) the level of endogenous timing uncertainty. For the collapsing thresholds we use two closed-form collapse functions: exponential and linear. Figure 3 depicts the threshold collapsing functions (assuming exponential collapse functions) that yielded the highest number of rewards for different response deadlines (for a given level of timing uncertainty) and for different levels of endogenous timing uncertainty (for a given deadline). As predicted by Frazier and Yu (2008), visual inspection of Figures 3A,B suggests that reward-maximizing threshold trajectories should nearly meet at the response deadline, and threshold collapsing should start earlier in the trial for shorter deadlines and higher levels of timing uncertainty. Our simulations showed very similar results when RR instead of “reward amount” is taken as an indicator of optimality. These results qualitatively mimicked the analytically derived functions found by Frazier and Yu (2008) for an objective function closely related to RR (see Methods).

To the best of our knowledge, the aforementioned predictions have not been directly tested by employing hard response deadlines (but see Balcı et al., 2011a for description of a pilot study). Neither has the relationship of 2AFC behavior under response deadlines been empirically related to the decision maker's level of endogenous timing uncertainty. The present study fills this empirical gap. Finally, we conducted further simulations to determine whether different levels of trial-to-trial variability of the core DDM parameters that might result from the introduction of the response deadlines can explain our data without alluding to dynamic (within-trial) threshold modulation. These simulations were necessary given that it is also possible to observe a reduction in conditional accuracy curves without any corresponding threshold modulation as suggested by Frazier and Yu (2008). Our simulations confirmed this possibility by showing that such declines in accuracy with RT in these conditional accuracy curves can emerge directly from Ratcliff's model without any within-trial collapsing of the threshold, as shown previously (Ratcliff and Rouder, 1998; Ratcliff and McKoon, 2008).

## Materials and methods

### Participants

Eleven adults (6 males and 5 females), aged between 18 and 24 years (*M* = 20) were recruited through announcements posted online at the daily newsletter of Koç University. One participant (male, aged 24) stopped attending experiments after the first session, and his data were discarded from all analyses. The experiment consisted of eight, daily, one-hour long sessions comprised of two Free Response (FR) sessions, four Deadlined Response (DR) sessions, and two Temporal Reproduction (TR) sessions in that order (see Procedure below). One participant missed a single DR session, and another participant missed the second TR session. The experiment was approved by the Institutional Review Panel for Human Subjects of Koç University and was in accordance with the principles of the Declaration of Helsinki. All participants provided written consent for their participation.

### Apparatus

All stimuli were presented on a 21″ LCD screen on an Apple iMac G4 computer, generated in Matlab using the Psychtoolbox Extension (Brainard, 1997; Pelli, 1997; Kleiner et al., 2007) on the SnowDots framework developed by Joshua Gold at the University of Pennsylvania. Participants sat at a distance of 58–63 cm from the screen, in a dimly lit room and provided their responses using a standard Apple iMac keyboard, and stereo noise-cancelling headphones worn throughout the experiment gave auditory feedback.

### Stimuli and procedure

#### Free response dot motion discrimination task

Stimuli were random dot kinematograms (see Gold and Shadlen, 2001; Shadlen and Newsome, 2001). These Random Dot Motion (RDM) stimuli consisted of a circular aperture of randomly moving white dots (3 × 3 pixels) on a black background, with a diameter of approximately 3 inches, centered on the screen. On each trial, 16% of the dots moved coherently in rightward or leftward direction (0 or 180 degrees respectively). The motion direction was assigned randomly with equal probability. Participants' task was to use the ‘Z’ or ‘M’ keys on the keyboard to report the direction of the coherently moving dots. Stimuli stayed on the screen until a response was given, at which moment they were terminated. Trials were separated by a response-to-stimulus (RSI) interval, sampled from a truncated exponential distribution with a mean of 2 s, a lower bound of 1 s, and an upper bound of 5.6 s. Correct responses were followed by an auditory beep indicating positive feedback, whereas no feedback was given for incorrect responses. This method of giving auditory feedback is standard in most 2AFC tasks, and has been shown to aid acquisition (e.g., Herzog and Fahle, 1997; Seitz et al., 2006), which was also the central purpose of our FR sessions (**Figure 6**). Premature/anticipatory responses (i.e., responses less than 100 ms after the offset of the stimulus) were penalized by a 4 s timeout, following a buzzing sound. Participants earned 2 kurus (approximately 1 cent) per correct response in experimental trials (excluding practice blocks), whereas no punishment was given for incorrect responses. The cumulative number of correct responses was presented on the screen every 10 trials in font size 12 (approximately 0.7 cm height). FR session consisted of a 2-min practice block, followed by eight 5-min test blocks, and a 4-min Signal Detection (SD) block. The data from these SD blocks were not used in this study.

#### Deadlined dot motion discrimination tasks

The DR sessions consisted of a 2-min practice block with FR trials, followed by one 5-min experimental FR block (same as the one described above), followed by two groups of four DR blocks, each group preceded by a 2-min practice block of the corresponding deadline (see below). Stimulus types and presentation schedules in DR blocks in these DR sessions were identical to those used in FR sessions, except for the assignment of either a short (800 ms) or a long (1000 ms) deadline to every trial in the block. In these DR trials, if the participant failed to respond before the pre-specified fixed deadline, the RDM stimulus disappeared, a buzzing sound was played (indicating a “late response”) and no reward was given for that trial. Otherwise, identical to the FR trials, the RDM stimulus disappeared upon a given response and a reward was given for correct responses.

After a 10 s intermission following the above-mentioned single 5-min FR block, and the 2-min practice block of DR trials, four 5-min experimental blocks with the same type of DR trials employing one of the deadlines (i.e., short or long) were presented. These blocks were followed by a 30-s intermission, after which the same order of practice and experimental blocks was presented, this time using the other deadline. Individual blocks were separated by a minimum break of 10-s, after which the participant made a button press to start the following block. The order of deadlines was randomized across the two halves of the eight DR blocks in each session. Identical with the FR sessions, two 2-min SD blocks were presented at the end of each session, and the data from these SD blocks were not used in this study.

These two hard deadlines were chosen based on the data collected from single session pilot testing with only the FR blocks. These data showed that the majority of participants' RTs ranged between 400 and 2500 ms, with a mean of 700 ms. Based on these data we chose two deadlines, the “easy” deadline of 1000 ms (on average 15% of the RTs were longer than 1000 ms; s.e.m. = 4.41) and the more “stringent” deadline of 800 ms (on average 28% of the RTs were longer than 800 ms; s.e.m. = 6.32). This way, we planned to have enough data from trials with RTs near the response deadlines. It can be argued that shorter deadlines might have made the task so difficult as to preclude strategic time-based decision-making. That said, we observed that participants sped up their free response RTs in the deadline blocks (**Figure 7**) and thus the deadline stringency was not as high as we intended during the study design. Nonetheless, the deadlines clearly exerted an effect on speed and accuracy relative to free responding, as we demonstrate below, and the two deadline durations should have been sufficiently discriminable from each other that a differential effect on behavior could have been expected. The ratio between 800 and 1000 ms constitutes a discriminable difference for humans; given a coefficient of variation (CV; Section Temporal reproduction task – static stimuli) of 0.12, the difference is over two standard deviations for the standard duration of 800 ms (Malapani and Fairhurst, 2002). This CV value is also consistent with earlier data (see Wearden, 2003).

#### Temporal reproduction task – static stimuli

The TR task consisted of the presentation of a stimulus for a specific duration, after which the participant tried to reproduce the same duration as accurately as possible by holding down the space button. The stimulus used in the first TR task was a 3 × 3 inch green square, placed in the middle of the screen. Each TR trial started with a button press after which the square was presented for a specific duration. The TR session started with a practice block of 9 trials using 3 randomly ordered target durations (i.e., 1.3, 2.3, and 3.3 s) with equal frequency. After the reproduced interval on practice trials, visual feedback was given by placing an approximately 1 cm white vertical line either to the left or right of a red reference line in the middle of the screen, representing the reproduced and given durations, respectively. The offset length of the white line was proportional to the difference between given and reproduced durations, whereas its location (left vs. right) showed under- or over-reproduction, respectively.

Nine 5-min test blocks of three target durations (1, 2.12, and 4.24 s), were presented in pseudo-random order following the practice trials. No feedback was given in test trials. The amount of money earned in each block was a function of the target duration, the average of absolute deviance scores for that block, and a maximum of 2.5 Turkish Liras that could hypothetically be earned with perfect performance (i.e., mean deviance score of 0), calculated using the following formula;

(2) $$\begin{array}{l} Total\;Earnings=Maximum\;Possible\;Amount\\ \;\times \;\left(1-Average\;Deviance\;Score/Target\;Time\right)\;\;\;\;\; \end{array}$$

Therefore, a smaller deviance score was required in a block of shorter target durations, compared to a block of longer to be-reproduced durations, in order to earn the same monetary reward.

The total amount earned was shown at the end of each block. Participants' endogenous timing uncertainties were quantified using reproduction data for each duration by dividing the standard deviation of reproduced durations by their mean. This is a statistical procedure for obtaining the CV of a dataset, and is used as an indicator of endogenous timing uncertainty, which is typically constant for different durations within an individual (Gibbon, 1977; Buhusi and Meck, 2005). The CV is an appropriate measure of timing uncertainty since when the CV is known, one can estimate the expected error of the same individual for other intervals (CV times *t*). Thus, many studies in the interval timing literature use CV as a measure of timing uncertainty (e.g., Gibbon, 1977; Balcı et al., 2011a).

#### Temporal reproduction task–RDM stimuli

These additional TR sessions were identical to the original TR session described above, except for replacing the static green square with a RDM stimulus identical to the one used in FR and DR sessions (i.e., dot motion stimulus with 16% coherence). The purpose in replacing the static stimulus with the RDM stimulus was to replicate as closely as possible the conditions in which the FR and DR sessions took place, since a TR task more similar to these 2AFC tasks could better capture the representation of attentional, as well as temporal, dynamics underlying the decision making process (see Zakay and Block, 1996). This in turn should lead to more accurate estimates of timing performance (i.e., CV) as manifest in the decision task and thus values that are more appropriate for generating threshold collapse predictions in DR sessions. In order to make sure that the motion direction was being attended to, participants were asked to report the direction of motion using the “Z” or “M” keys in 20% of the trials, following the time reproduction. “Total Earnings” (Equation 2) were multiplied by the proportion of accuracy in reporting the direction of motion in each block.

Since the error rate in direction judgments would inevitably decrease the total amount earned in these TR sessions compared to those using the static stimuli, the maximum possible amount that could be earned per block was increased from 2.5 to 3 Turkish Liras. Each TR task (i.e., with static or RDM stimuli) lasted for a single session. The TR testing was shorter than the 2AFC tasks because estimating temporal accuracy and precision does not require as large of a dataset as one needs for the DDM fits and conditional accuracy curves.

### Data analysis

#### Quantifying declining accuracy with time

In order to quantify a possible decline in accuracy as time elapsed within trials, accuracy levels were calculated for each 50 ms RT bin, forming the conditional accuracy curves. Bins with less than 4 data points, as well as RTs above 5 s, were excluded from all further analyses. The exclusion criterion for bin size was based on *post-hoc* analyses of the data, especially for the last two RT bins (i.e., at around the deadline), which generally contained less data points than the ones that corresponded to shorter RTs. Our analysis showed that nine participants had at least 4 data points in the last RT bin in the short deadline condition, whereas this number declined to four participants in the long deadline condition. Since the accuracy at and near the deadline was of high relevance to this study, we set our exclusion criterion to allow for involving these participants' RT data in further analyses. Note that our original choice of the specific response deadlines based on free response RT distributions aimed for more data points to fall in these later bins.

A conditional accuracy curve allows us to determine the RT bin where a decline in accuracy starts, as well as the rate of this decline. In order to define the specific point where the accuracy trend changes, we found the RT bin at which the sum of squared errors of two piece-wise linear fits to data before and after that point (a.k.a. the knot) is minimized. This was achieved by running an algorithm which fits the piece-wise linear functions to data by using each RT bin as a putative knot location where the first linear function is “latched on” to the second one. Specifically, the algorithm constrains the intercept of the second linear fit to be the last value of the first fit, forming two connected lines. Since the last data point of the first fit affects the fit of the second line by slightly modifying its slope, the algorithm runs in both forward and reverse directions, ensuring that it finds the knot location where the total error of the piece-wise fit is minimal, regardless of which of the two slopes is modified. The purpose of using this algorithm was to quantify the onset (i.e., inflection point), as well as the slope of a possible decline in accuracy with RT separately for two different deadlines. The correlations of these two values (i.e., onset & slope) with timing uncertainty were later calculated (Section Effect of Deadlines on Response Time and Accuracy) in order to test if higher levels of timing uncertainty predicted an earlier onset of decline in accuracy characterized by a lower (as opposed to a steep) negative slope.

#### Optimal threshold collapse simulations

We conducted simulations in order to approximate the optimal threshold collapsing trajectories for different deadline durations (800, 1000, and 1200 ms) and six linearly increasing levels of endogenous timing uncertainty (i.e., CV), using two different closed-form collapse functions (i.e., exponential and linear). Below we describe the details for the exponential threshold collapse function, but the same procedure applies to the linear collapse function as well. Although our response paradigm employed only two deadline durations (800 and 1000 ms), we have also tested the 1200 ms deadline in these simulations. For the objective function analyzed by Frazier and Yu (2008)–which may approximate but is not identical to RR—analytically optimal collapse functions look much like our exponentials.

In order to find the exponential threshold collapsing trajectory that maximizes the number of rewards for a given deadline and a given timing uncertainty, we first constructed a total of 101 threshold trajectories with 0.01 second increments, separately for each CV value. The following formula was used to construct an exponential curve:

(3) $$a=\left(Asymptote+\left(Starting\;Point\;-\;Asymptote\right)\times e^{\left(-c*t\right)}\right)\;\;\;$$

where *Asymptote* was set at 0.1 for the upper threshold, Starting Point was set at 0, *c* represented the rate of exponential decline (i.e., as a proxy for temporal discriminability), and *t* is time. The resulting curve was then flipped on its y-axis to construct the upper threshold. This mirror image of the upper threshold was used as the lower threshold (Figure 3).

All thresholds collapsed exponentially with time to the starting point of evidence accumulation (Figures 3A,B). The upper and lower thresholds with the earliest evaluated collapse onset met well before the shortest deadline (i.e., 800 ms), and the thresholds with the latest evaluated collapse onset met well after the longest deadline (i.e., 1200 ms). The presumed effect of the timing uncertainty was implemented by changing the exponential decay parameter (*c*; e.g., steeper collapse for higher temporal discriminability due to lower timing uncertainty).

For each response deadline, we defined the optimal threshold trajectory as the one (out of 101 per CV) that yielded the greatest number of rewards out of 10^6^ drift-diffusion simulations. In line with our experimental paradigm, in these simulations RTs longer than the deadline duration were not assigned any reward. The drift diffusion processes were simulated based on Equation 1. The drift rate was set to 0.1, the noise coefficient was set to 0.1, the starting point was set to 0 and non-decision time was set to 0. The two decision thresholds were set to −0.1 and 0.1 at trial onset. For simplicity, the core parameters were not allowed to vary between trials. The results of our simulations supported Frazier and Yu's formulation; the optimal thresholds for a given deadline and a given CV were the ones which nearly reached the starting point at the response deadline even with closed-form collapse functions (Figure 3A). These simulations also suggested that higher timing uncertainty requires an earlier onset of threshold collapsing, so that the upper and lower decision thresholds are ensured to meet virtually at the deadline.

We have also calculated the optimal threshold collapse trajectories by setting the criterion for optimality as the highest RR instead of the highest amount of expected reward (Figure 3B). The RR for each collapse trajectory was calculated by dividing the mean accuracy by the mean RT. In calculating the RR, late responses (i.e., those beyond the deadline) were given a value of 0 for accuracy (i.e., they were counted as error trials). RT was defined as “DT + RSI + *T*_*er*_”for trials with RTs faster than the deadline, and “deadline + RSI” for trials where RTs were slower than the deadline. Using values for the RSI and *T*_*er*_ very close to the ones derived from our experimental paradigm, calculated the expected RR for each collapse trajectory and found that, similar to those in Figure 3A, optimal thresholds for a given CV were the ones that roughly collapsed to the starting point near the deadline (Figure 3B).

**Figure 4:**
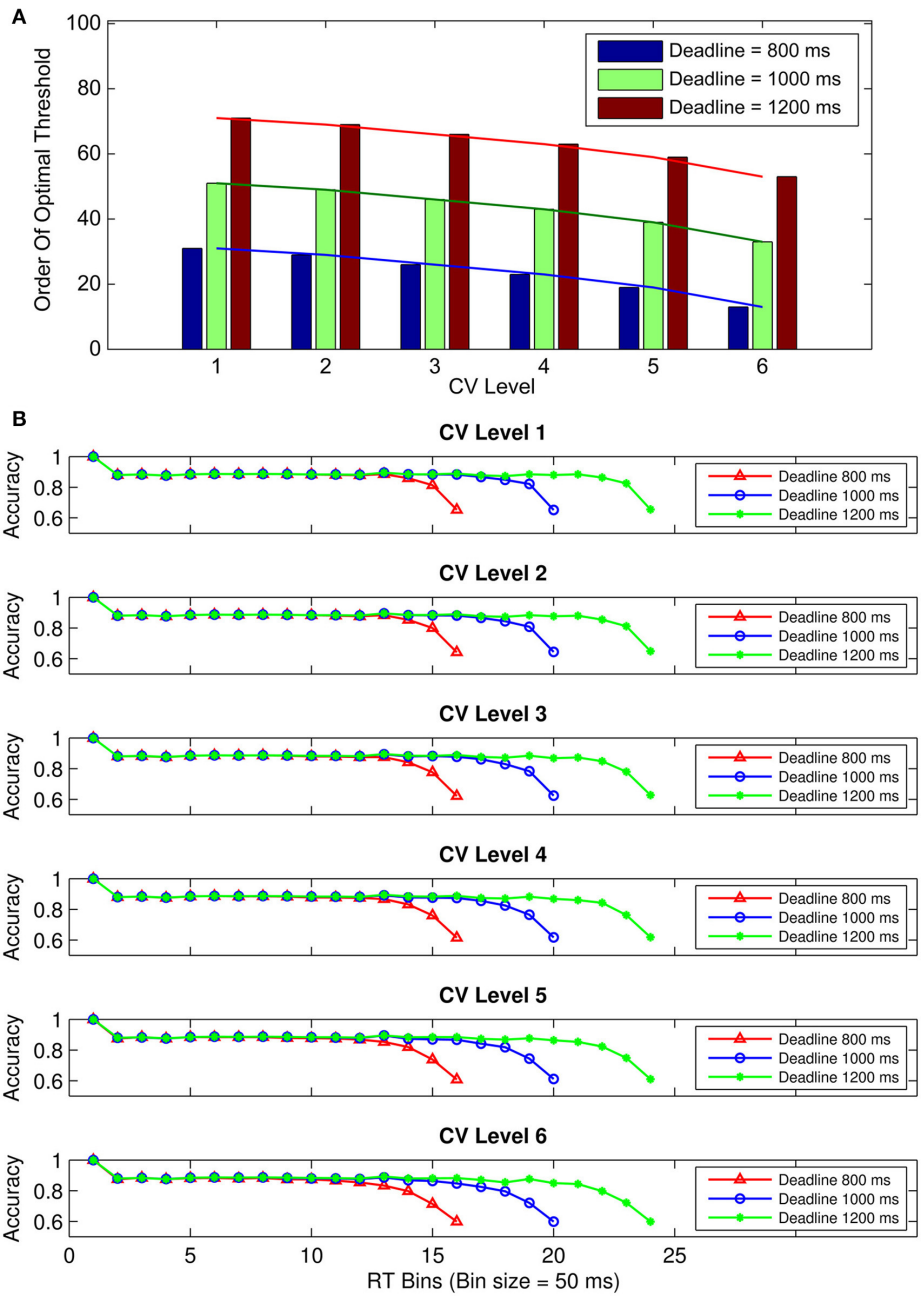
**(A)** Bar graphs depicting the order of the optimal threshold collapse trajectories (out of 101 thresholds with 0.01 s increments) selected from the family of exponential decline functions for six hypothetical levels of timing uncertainty. Lines connect the bars. **(B)** Conditional accuracy curves for the six CV conditions, shown separately for the three response deadlines. Red lines represent the conditional accuracy curves for the short deadline (800 ms), blue lines for the medium deadline (1000 ms), and green lines for the long deadline (1200 ms). Both **(A,B)** are based on expected total reward as the optimality criterion.

Visual inspection of Figure 4A shows that the order of the optimal threshold (i.e., the order of a given threshold among the 101 thresholds tested with 0.01 s increments) increases with longer deadlines for a given CV, in addition to decreasing with higher CVs for a given deadline. Additionally, conditional accuracy curves were plotted for the six hypothetical CV levels, separately for the three deadline durations (Figure 4B). The level of CV (i.e., the level of endogenous timing uncertainty) was increased or decreased by decreasing or increasing the rate of exponential decline (the *c* parameter in Equation 3), respectively. Visual inspection of Figure 4B suggests that accuracy in our simulations declines with time for all levels of CV. However, contrary to our expectations, accuracy never fully reaches 50% (chance level) in these curves. Both Figures 4A,B were constructed based on expected total reward as the optimality criterion.

**Figure 5:**
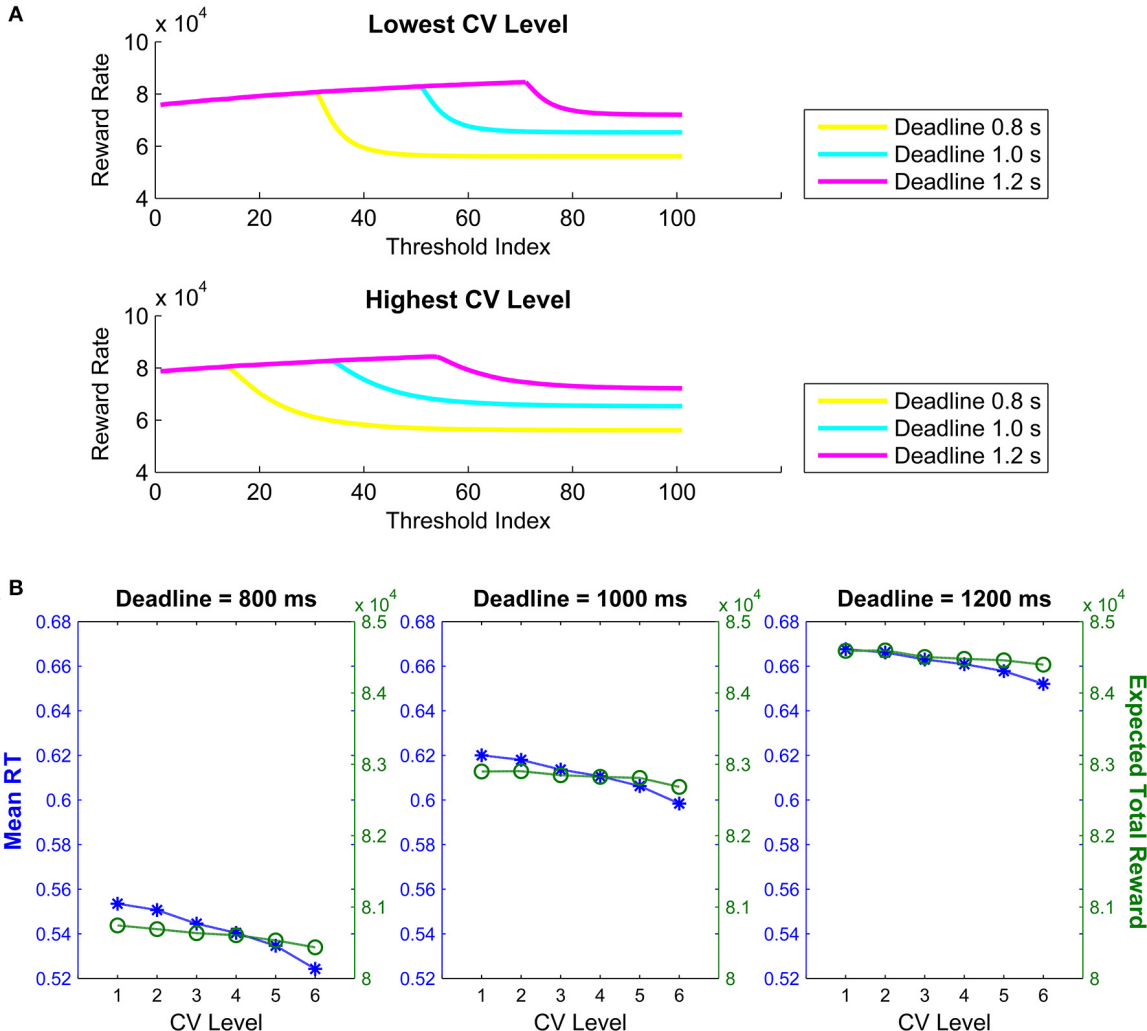
**(A)** Expected total reward amount for the highest and lowest CV levels as a function of the order of threshold among the 101 thresholds tested (here defined as “Threshold Order”). **(B)** Mean response times and expected total reward amounts as a function of six levels of CV defining six exponential threshold collapse trajectories for the short (800 ms), medium (1000 ms) and long (1200) simulated deadlines. Both **(A,B)** are based on expected total reward as the optimality criterion.

Finally, Figure 5A shows the expected total reward curves for all 101 collapse functions constructed with the lowest and the highest CV levels (out of the six CV levels) for the three deadline durations. Visual inspection of Figure 5A suggests that the expected total reward steadily increases with the order of exponentially collapsing thresholds, and sharply declines immediately following the deadline. Additionally, Figure 5B shows the mean RTs and expected total rewards predicted for optimal threshold trajectories as a function of CV, separately for the three deadlines. Figure 5B suggests that with increasing timing uncertainty (i.e., CV level), both the mean RT and the expected total reward decline. See Supplementary Material for the linear threshold collapse results.

## Results

### Accuracy and response time in the free response conditions

**Figure 6:**
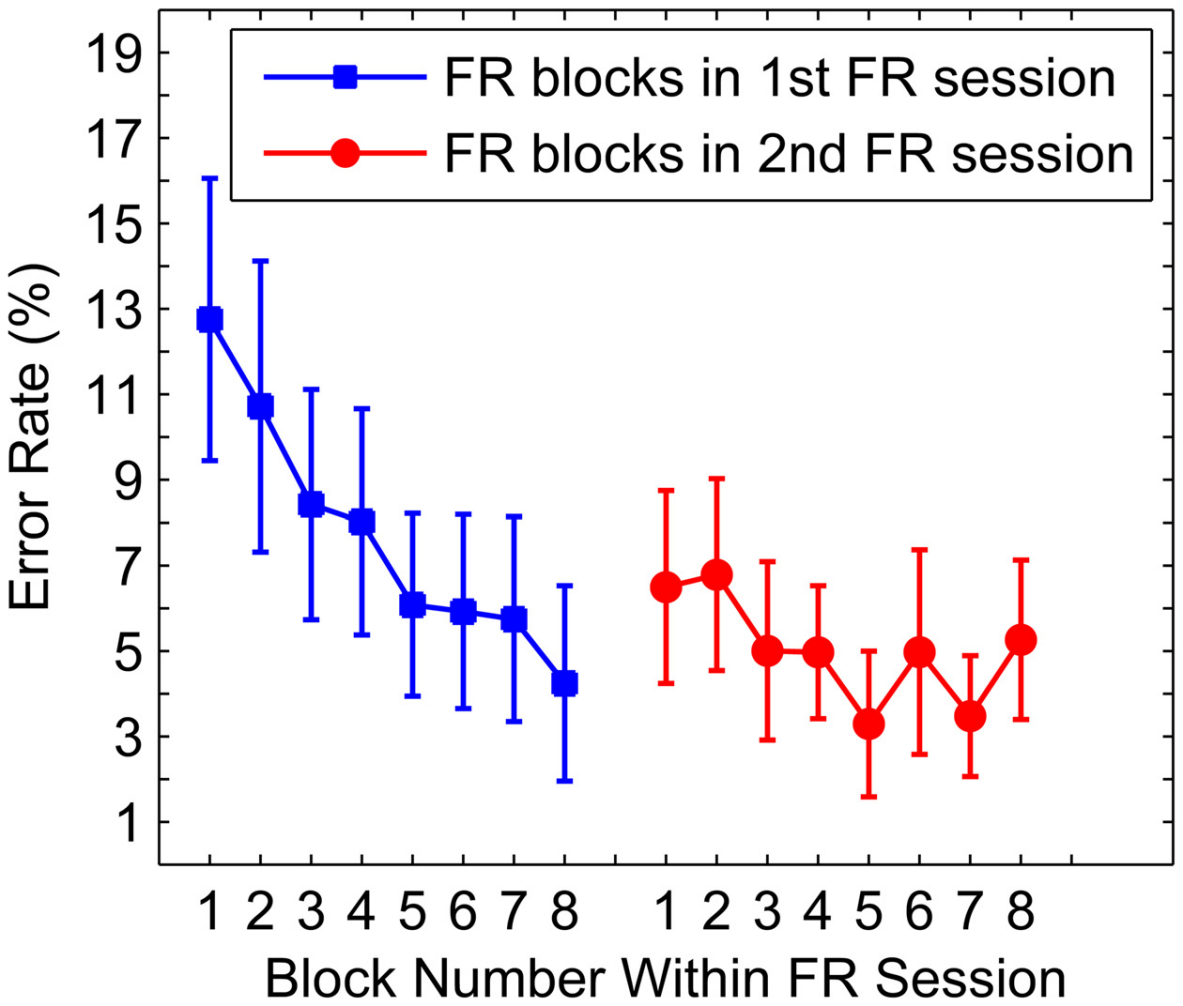
**Mean error rate as a function of FR block**. Mean error rates per FR block in the first two sessions (i.e., FR sessions). Blue squares correspond to FR blocks in the first FR session and red circles correspond to FR blocks in the second FR session. Error bars denote the standard error of the mean.

The data from the two FR sessions showed that the participants' error rates declined from a mean of 10% in the first 4 blocks of the first FR session, to a mean of 4.3% in the last 4 blocks of the second FR session [*t*_(9)_ = 3.1, *p* < 0.05; Figure 6] suggesting that the FR sessions were successful in training the participants on the RDM discrimination task. Additionally, the RTs showed a similar decline with increasing blocks, with a mean of 0.94 s in the first 4 blocks of the first FR session, to a mean of 0.75 s in the last 4 blocks of the second FR session, however, this difference failed to reach significance (*p* > 0.05). RTs between the first and second halves within the two FR sessions did not differ significantly (both *p*s > 0.05), excluding the potential role of factors such as an increased fatigue or inattention toward the end of a test session.

**Figure 7:**
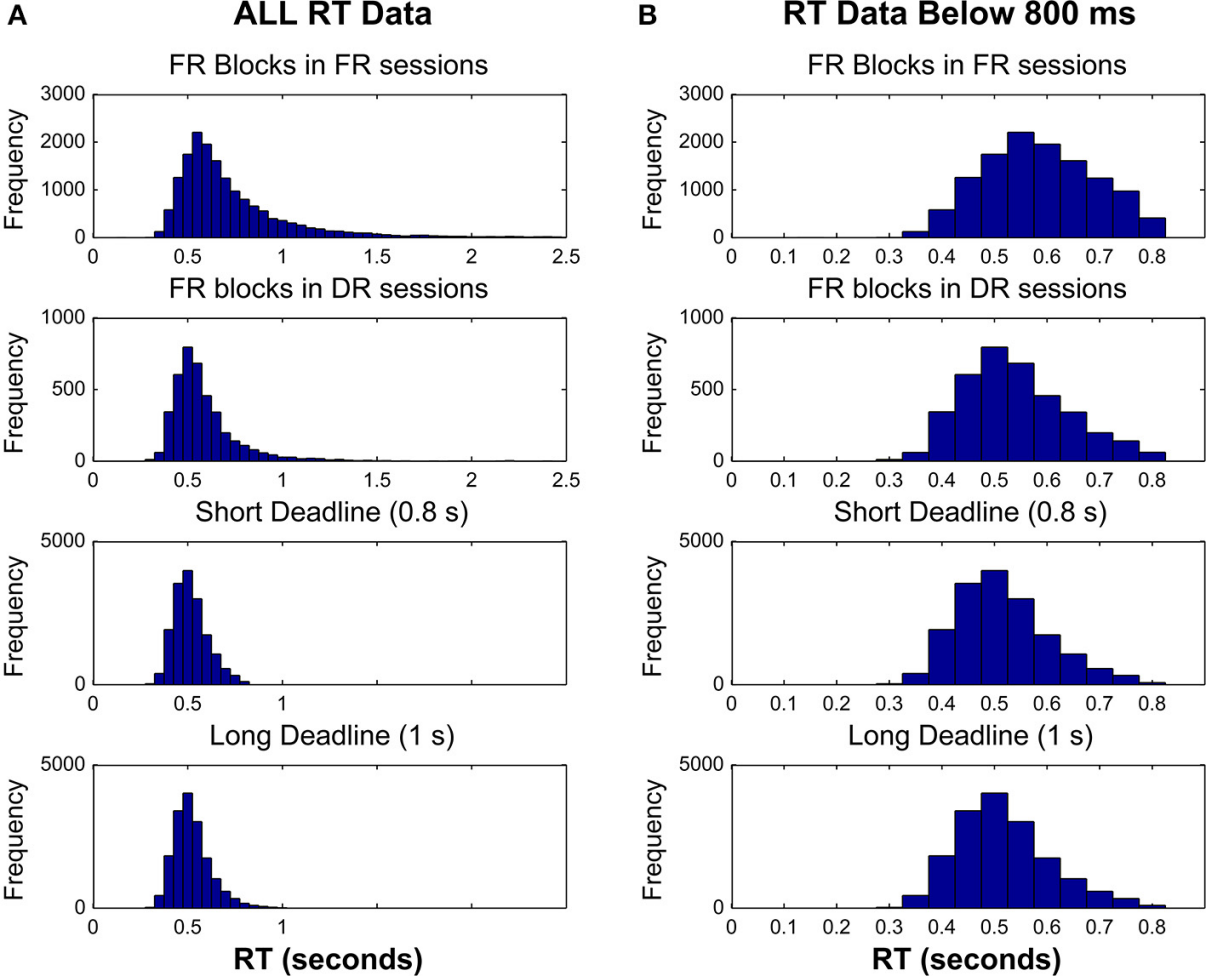
**Response time distributions for FR blocks in FR sessions, FR blocks in DR sessions, Short DR blocks in DR sessions and Long DL blocks in DR sessions**. RT data pooled across participants are plotted either **(A)** without an upper limit, or **(B)** with an upper limit of the short deadline duration (800 ms).

Figure 7 shows the RT distributions in the FR blocks in FR sessions, FR blocks in DR sessions, and the two deadline blocks in the DR sessions. Figure 7 shows the plots either of all RTs pooled across participants (Figure 7A), or RTs below the short deadline duration (Figure 7B). A mean of 844.85 (s.e.m. = 20.1) trials were completed in FR blocks in FR sessions, whereas this number was 105.2 (s.e.m. = 1.24) in FR blocks in DR sessions, 433.88 (s.e.m. = 2.27) in Short Deadline blocks in DR sessions, and 432.48 (s.e.m. = 2.82) in Long Deadline blocks in DR sessions.

### Effect of deadlines on response time and accuracy

In order to determine whether introducing a deadline for responding was successful in modifying behavior, we first compared the mean RT values obtained by pooling data from both FR sessions, the 4 DR sessions (separately for the short and long deadline conditions), and the single FR blocks presented at the start of each DR session for each participant. A one-way repeated measures ANOVA was conducted to compare the effect of response time limitations on mean RT in four conditions; two free response (i.e., FR blocks in FR sessions and FR blocks in DR sessions) and two deadline (i.e., short & long) conditions. Since response deadlines act as a procedural censoring point for slower RTs, only the RT values up to the short deadline (800 ms) were compared in all conditions. Our analysis indicated a significant effect of different experimental conditions on the RTs, *F*_(3,6)_ = 32.78, *p* < 0.001. Tests of six pair-wise comparisons were conducted using Holm-Bonferroni adjusted alpha levels. These comparisons showed that RTs in FR blocks in FR sessions (*M* = 602 ms) were significantly longer than both the short deadline (*M* = 519 ms, *p* < 0.001) and the long deadline (*M* = 525 ms, *p* < 0.001) conditions, as well as the response times of FR blocks in DR sessions (*M* = 548 ms, *p* < 0.001). The difference between the RTs in the two separate deadline conditions and the FR blocks in DR sessions did not reach significance (both *p*s > 0.05). However, when no correction was applied for multiple comparisons, the mean RT differences between FR blocks in DR sessions and the two separate deadline conditions reached significance (both *p*s < 0.05).

In order to further test if introducing a short vs. long deadline was effective, we compared the number of missed deadlines for each deadline condition. A mean of 1.68% of deadlines were missed in the short deadline condition (s.e.m. = 0.35), whereas this percentage declined to a mean of 0.36% in the long deadline condition (s.e.m. = 0.09). A paired samples *t*-test revealed that the percentage of missed deadlines was higher for the short deadline condition, compared to long deadline condition *t*_(9)_ = 4.5, *p* < 0.001. In other words, participants as expected were more likely to miss the deadline in the short DL conditions compared to the long DL conditions. The hypothetical percentage of missed deadlines was computed for the RT distributions of the FR blocks in DR sessions by calculating the percentage of the data above the RTs corresponding to the two deadlines separately. A mean of 9.13% of the trials (s.e.m. = 3.02) had RTs above the short deadline duration (i.e., 800 ms), whereas a mean of 3.26% of the trials (s.e.m. = 1.15) had RTs above the long deadline duration (i.e., 1000 ms). Matched-sample *t*-tests showed that the percentage of RTs above the short deadline duration in FR blocks in DR sessions was significantly higher compared to the percentage of missed deadlines in the short deadline condition *t*_(9)_ = 2.89, *p* < 0.05. Similarly, the percentage of RTs above the long deadline duration in FR blocks in DR sessions was significantly higher compared to the percentage of missed deadlines in the long deadline condition *t*_(9)_ = 2.81, *p* < 0.05. These results point at the effect of response deadlines on RTs.

An additional One-Way repeated measures ANOVA was conducted to compare the effect of four experimental conditions on overall accuracy, using accuracy data corresponding to RTs below 800 ms (again due to the procedural censoring factor). There was a significant effect of experimental condition on accuracy, *F*_(3,6)_ = 22.59, *p* < 0.001. Tests of six pair-wise comparisons conducted using Holm-Bonferroni adjusted alpha levels revealed that, whereas the accuracy in FR sessions (*M* = 0.96) and FR blocks in DR sessions (*M* = 0.94) did not differ significantly from each other (*p* > 0.05), both accuracy means differed significantly from the short (*M* = 0.90, both *p*s < 0.001) and long deadline (*M* = 0.90, *p*s < 0.001) conditions. Mean accuracy in the two deadline conditions did not differ significantly (*p* > 0.05).

The effect of four experimental conditions on overall accuracy were also compared using all data, without excluding those above 800 ms. There was a significant effect of experimental condition on accuracy, *F*_(3,6)_ = 8.07, *p* < 0.001. Tests of six pair-wise comparisons conducted using Holm-Bonferroni adjusted alpha levels revealed that, whereas the accuracy in FR blocks in FR sessions (*M* = 0.94) and FR blocks in DR sessions (*M* = 0.93) did not differ significantly (*p* > 0.05), mean accuracy in FR blocks in DR sessions differed significantly from both the short (*M* = 0.90) and long deadline (*M* = 0.90) conditions (both *p*s < 0.001). The mean accuracy in the two deadline conditions did not differ significantly either from each other or from the mean accuracy in FR blocks in FR sessions (all *p*s > 0.05). However, when no correction was applied for multiple comparisons, the mean accuracy differences between FR blocks in FR sessions and the two separate deadline conditions reached significance (all *p*s < 0.05).

We analyzed within block RTs in both deadline conditions to verify that inattention/fatigue did not set in toward the end of a 5-min block, possibly resulting in slower RTs toward the end of a block. For this purpose, we first calculated individual participants' mean RTs for each trial order in separate deadlined blocks across all DR sessions, for the two deadline conditions. For instance the mean RT for trial number 14 in the second block of all short deadlined DR sessions was calculated by taking the mean of all RTs corresponding to the 14th trial in the second blocks of the short deadlined DR sessions and so on. For later trials where some blocks did not have RT data due to unequal number of trials per block, mean RT was calculated by using available data only. Given that there were four blocks in each deadline condition per session, this procedure resulted in four sets of mean RTs per participant, which were fit by a linear regression using a least-squares method. It was reasoned that an increase in RTs over the course of a block of trials should manifest itself as a positive slope of a linear fit to data. A total of eight one-sample *t*-tests were conducted (four for each deadline condition) in order to determine whether the slopes of the linear fits were different from 0. None of the slopes were significantly higher or lower compared to the test value of 0 (all *p*s > 0.05), suggesting that RTs did not increase or decrease toward the end of a test block.

Finally, we wanted to see if error trials were more likely to occur in the first half or the second half of a DR block, due to possibly increasing fatigue or inattention. Using the same method described above, we calculated individual participants' mean accuracies in the first and the second halves of each block, separately for the two deadline conditions. Eight paired sample *t*-tests were conducted to compare accuracy in the two halves of each block in the two deadline conditions (i.e., four *t*-tests for each condition). None of the differences were significant, suggesting that accuracy did not decline toward the end of a deadlined test block (all *p*s > 0.05).

### Accuracy at deadline

In order to see if it declined to chance level at the deadline, accuracy in the last 50 ms RT bin was calculated for both deadline conditions. Nine participants had valid data (i.e., more than 4 data points) in this RT bin in the short deadline condition, with a mean accuracy of 78.4% (s.e.m. = 3.6%), whereas 4 participants had data in the last bin in the long deadline condition with a mean of 75.6% (s.e.m. = 5.8%). Of those with valid data in the last bin, no participant's accuracy fell below 63% in the short deadline condition, whereas the lowest accuracy in the last bin was 60% in the long deadline condition. A Wilcoxon signed ranks test indicated that accuracy in the last RT bin in the short deadline condition (*Mdn* = 0.76) was significantly higher than a hypothetical value of 0.5 (*Z* = 45, *p* < 0.05), whereas this difference did not reach significance for the last RT bin in the long deadline condition (*Mdn* = 0.78, *p* > 0.05).

### Piece-wise linear fits of conditional accuracy curves

**Figure 8:**
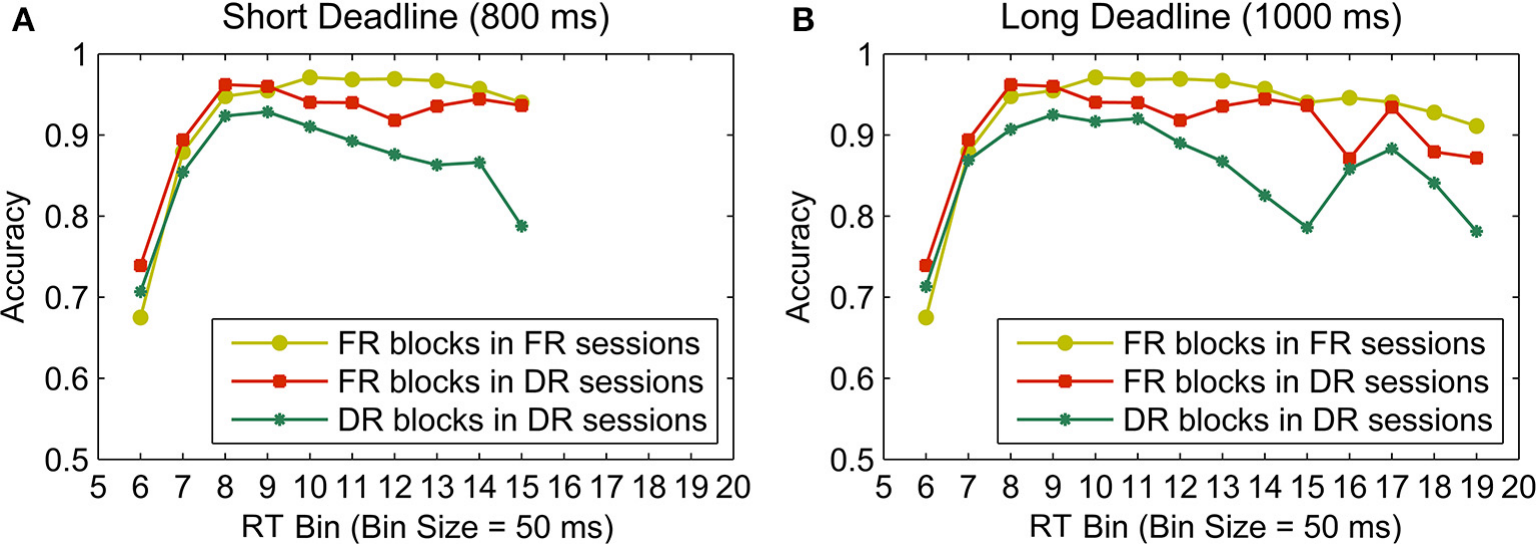
**Conditional accuracy curves for the FR blocks in FR sessions, FR blocks in DR sessions, and DR blocks separately for the short (A) and long (B) deadlines (800 and 1000 ms, respectively)**. The conditional accuracy curves for the two FR blocks are identical between two columns up to the 15th RT bin. Data were pooled across participants.

**Figure 9:**
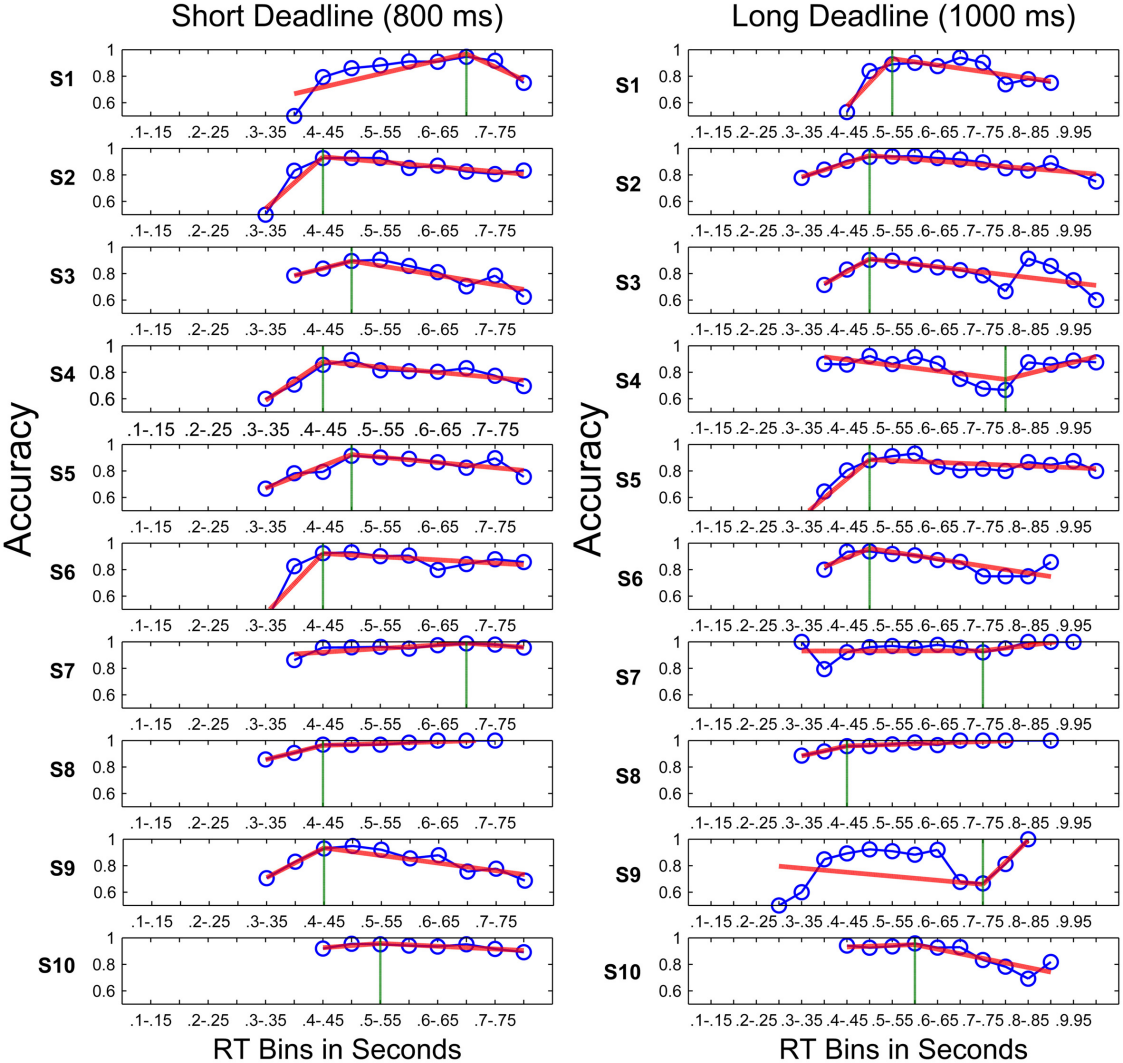
**Piece-wise linear fits (red lines) to conditional accuracy for the DR blocks (blue lines with circles) of all participants in the short deadline (left) and long deadline (right) conditions**. Vertical green lines indicate inflection points.

Figure 8 shows the conditional accuracy curves plotted for each condition by pooling data across participants. The analysis using piece-wise linear fits was also based on each participant's data expressed as conditional accuracy curves (Figure 9). The knot locations (defined in terms of RT bins) of the piece-wise linear fits to these data and the slopes of the best fit lines were calculated using the algorithm described in the Methods Section, in order to quantify the onset, as well as the rate of a potential decline in accuracy with time. Figure 9 shows fits to individual participants' data. A total of 9 out of 10 participants had declining accuracies after the inflection point (i.e., knot location) with time (i.e., negative slope) in the short deadline condition, whereas 6 had declining accuracies after the inflection point in the long deadline condition. Two one sample *t*-tests were conducted in order to compare the slopes of the second line for the two deadline conditions to the slope of “0” (i.e., no decline in accuracy with time). Although, the slopes in the short deadline condition (*M* = −0.3) differed significantly from 0 [*t*_(9)_ = 2.84, *p* < 0.05], this difference failed to reach significance in the long deadline condition (*M* = 0.01, *p* > 0.05). The insignificant difference remained for the long deadline condition when the data from participant 9 with a bad fit were not included in the analysis.

### Temporal uncertainty and conditional accuracy curves

**Figure 10:**
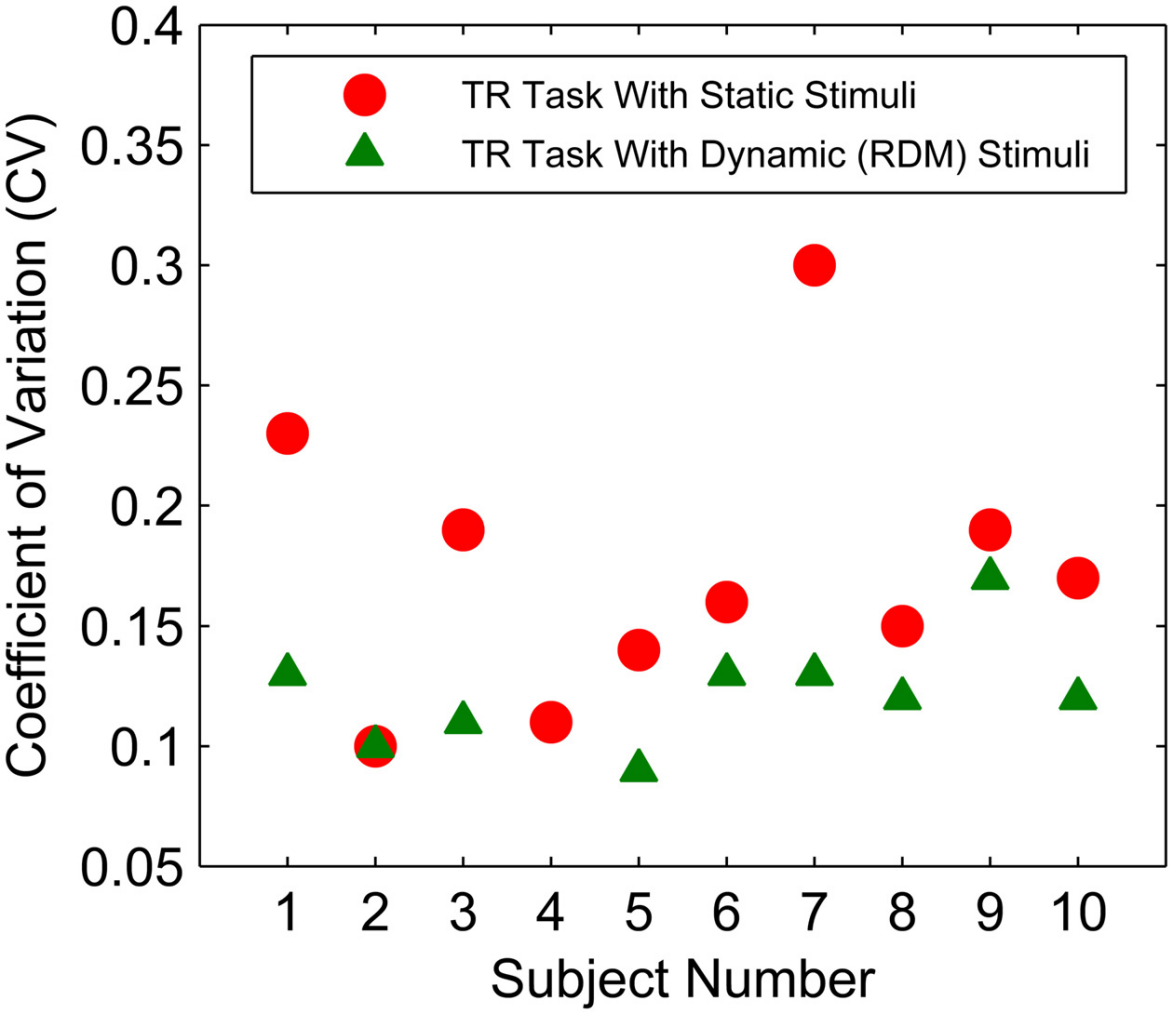
**CV values obtained in the temporal reproduction task with two different types of stimuli (red circles for static stimuli & green triangle for dynamic stimuli) for each subject**. Subject 4 did not participate in the second TR session with dynamic stimuli.

Coefficient of variation values for each participant were calculated for both TR tasks by taking the average of all CVs for the three target durations (see Methods Section; Figure 10). Mean CV values obtained from the first TR task using static stimuli were significantly higher compared to CVs obtained from the second TR task using RDM stimuli [*t*_(9)_ = 3.97, *p* < 0.01], which may reflect a practice effect since the first TR task always used static stimuli or the specific stimulus effect. A potentially significant correlation between RT and CV was examined. Neither of the CV values obtained from the two TR tasks correlated significantly with mean RTs in the FR or DR conditions (all *p*s > 0.05).

While the positive correlation between CVs in the TR task with static stimuli and the knot location of the piece-wise fits to RT data in the short deadline condition reached significance [*r*_(8)_ = 0.85, *p* < 0.01, two-tailed], the same CVs did not correlate with the knot locations in the long deadline condition (*p* > 0.05). Conversely, the CVs obtained in the TR task with dynamic stimuli were positively correlated with the knot location of the piece-wise fits in the long deadline condition [*r*_(7)_ = 0.72, *p* < 0.05, two-tailed], whereas they did not correlate with those knot locations in the short deadline condition (*p* > 0.05). Neither of the CVs correlated with the slopes of the first or second line of the piece-wise linear fits (both *p* > 0.05).

As can be seen in Figure 9, participant number 9 had a visibly bad piece-wise linear fit to his/her conditional accuracy curve in the long deadline condition. Therefore, the same correlations were also calculated by excluding this participant's data in the long deadline condition. While the correlation between CVs in the TR task with static stimuli and the knot location of the piece-wise fits in the long deadline condition remained insignificant (*p* > 0.05), the correlation between CVs in the TR task with dynamic stimuli and the knot location in the long deadline condition also failed to reach significance when calculated by excluding this participant's data. Excluding this participant's data also did not result in a significant correlation between CVs and the slopes of the first or second line of the piece-wise linear fits to the long deadline condition (all *p*s > 0.05). None of these results support the optimal performance predictions, since we expected participants with higher CVs to start reducing their accuracy earlier (under the threshold collapsing assumption). If anything we observed the opposite relationship with the CVs in TR task with static stimuli in the short deadline condition, and the CVs in TR task with dynamic stimuli in the long deadline condition. When the data only from the participants with a negative slope in the second line of the piece-wise linear fits were taken into consideration, none of the correlations between either of the CVs and the knot locations, or between the CVs and the slopes of both the first and second line of the piece-wise linear fits reached significance (all *p*s > 0.05).

Even though we had a minimum number of data points per RT bin used in forming the conditional accuracy curves, investigating the declining accuracy using binned RTs may be misleading in the sense that some bin accuracies calculated with fewer yet highly accurate/inaccurate trials may be artificially inflated / deflated. In other words, the binning methodology may fail to accurately represent the dynamics of a declining accuracy with time, since it entails estimating accuracies for a specific time period from the average of sometimes a very limited number of data points. Therefore, we also calculated peak accuracy by taking the cumulative average of accuracy with increasing time (i.e., RT), and correlated the location of these peaks in time with CV values. This was achieved by first sorting RTs for each trial in increasing order and then forming an “accuracy vector” by coding 0 for error trials and 1 for correct trials corresponding to each RT value. Cumulative accuracy was then calculated for each trial by taking the average accuracy of all trials with RTs at and below that trial, which formed a cumulative average accuracy curve. Consistent with the findings reported above, the RTs at which the cumulative average of accuracy peaked did not correlate significantly with the CVs estimated from either TR task (both *p*s > 0.05). These results further supported the above-mentioned results obtained by using the RT binning approach, further suggesting that even if participants collapsed their decision thresholds, they did not take into account their endogenous timing uncertainties.

Finally, in order to see if there was a bias toward over- or underestimating the durations/deadlines additional analyses were conducted. Normalized mean reproduction durations of all participants were first calculated by dividing the mean reproduction duration by the target duration. This was done separately for all three durations (1–2.12–4.24 s) tested in the two TR session types (static or dynamic stimuli). Six one-sample *t*-tests were conducted using “1” as test value for accurate normalized performance. Only the 1 s test duration in the dynamic stimulus condition (*M* = 1.31, s.e.m. = 0.0.06) was systematically overproduced by the participants [*t*_(8)_ = 4.73, *p* < 0.001], suggesting that subjects tended to underestimate 1 s of dynamic stimulus presentation. This result suggests that if thresholds did in fact collapse with time, this collapse may have started declining later than optimally, since participants were underestimating the deadlines. In order to test this possibility, the correlation between the mean reproduction duration of 1 second (separately in the TR tasks using static & dynamic stimuli), and the knot location, as well as the slope parameter of the conditional accuracy curves was calculated. This procedure was also repeated by excluding the long deadline data of participant ID 9. None of these correlation coefficients reached significance (all *p*s > 0.05).

### Drift-diffusion model simulations

Since we observed accuracy reduction within trials for some participants in DR sessions, it is important to address whether models with fixed parameters within trials can account for this pattern. Thus, we tested if observed reduction in accuracy as a function of RTs could be due to factors other than collapsing thresholds. For this purpose, individual participants' data between FR blocks in DR sessions were fit by the extended DDM (i.e., allowing for inter-trial variability parameters, *all variability parameters* > 0, and also allowing for starting point bias) using the diffusion model analysis toolbox (DMAT) (Vandekerckhove and Tuerlinckx, 2008). These parameters were then averaged across participants in order to obtain a representative set of parameters that could be used for DDM simulations to follow.

The following mean parameters were obtained; decision boundary (*a*) = 0.1214, non-decision related delays (*T_er_*) = 0.4419, drift rate variability (*Var*(*v*)) = 0.1922, starting point (*z*) = 0.0608, starting point variability (*Range*(*z*)) = 0.0547, non-decision time variability (*Range* (*T_er_*)) = 0.1668, and drift rate (*v*) = 0.4447. Data from FR blocks in DR sessions were used instead of FR blocks in FR sessions to estimate the DDM parameters because they represent performance that is closer to steady-state.

**Figure 11:**
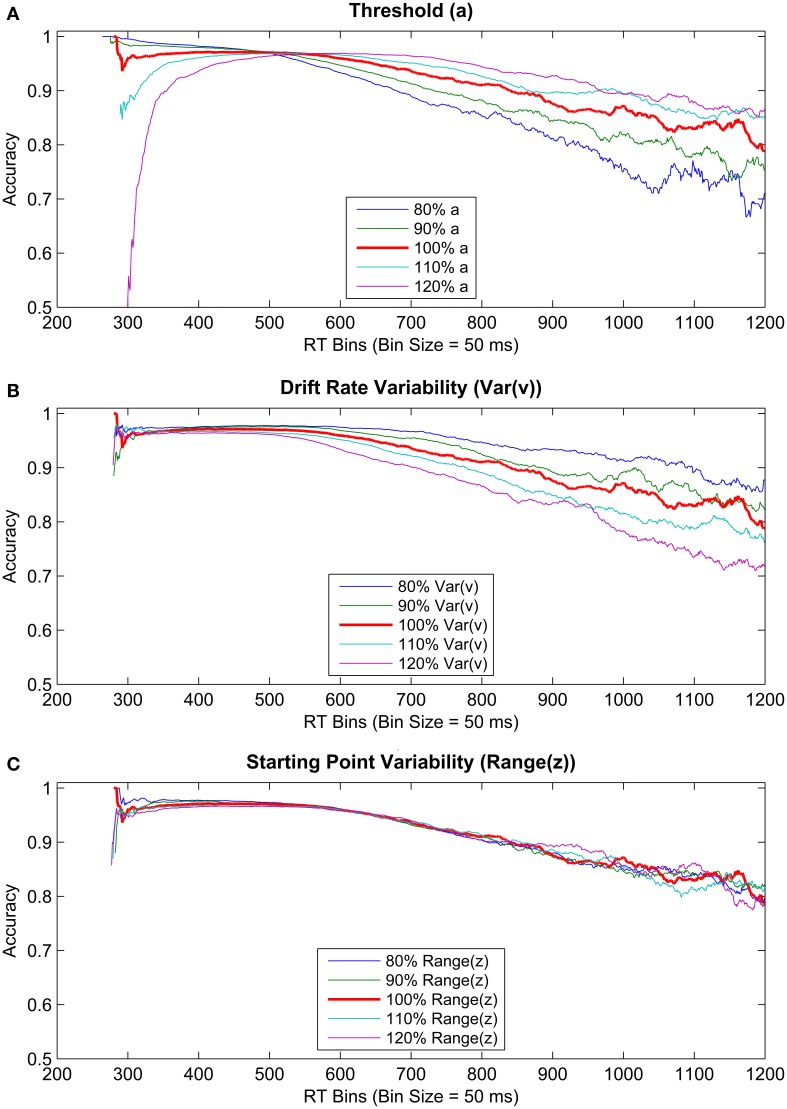
**Conditional accuracy curves gathered from simulated data**. RT bin size of 50 ms was used for plotting. Three parameters were systematically modified by increasing and decreasing their values by 20 and 10%. The parameters manipulated were **(A)** threshold (top panel), **(B)** drift rate variability (middle panel), and **(C)** starting point variability (bottom panel).

Using these DDM parameters, we simulated three sets of 10^6^ data points using DMAT's simulation feature, in which either of the threshold (*a*), drift rate variability (*Var*(*v*)), or the starting point variability (*Range*(*z*)) parameters were increased or decreased by 10 and 20% (depending on the condition; see Figure 11). Therefore, each set contained five levels of its corresponding parameters. This procedure aimed to investigate if changes unrelated to within-trial threshold collapsing might also lead to decreasing accuracy levels with slower RTs. These specific parameters were chosen for incrementing/decrementing because large/small values of these parameters are known to lead to longer/shorter RTs for incorrect choices (Ratcliff and Rouder, 1998; Ratcliff and McKoon, 2008). Specifically, larger values of threshold and drift rate variability parameters lead to slower error RTs, whereas a larger variability in starting point should present itself as faster RTs for error trials (Ratcliff and Rouder, 1998; Ratcliff and McKoon, 2008). Such response patterns formed by slower responses for error trials compared to correct ones cannot be explained by the pure DDM when it is unbiased toward one threshold over the other (Laming, 1968). Importantly for our purposes, if error trials are slower than correct trials, this pattern automatically implies a declining conditional accuracy curve. In other words, the decline in accuracy observed in our data may not necessarily be a behavioral manifestation of a collapsing decision threshold (*a*), but instead may result from changes in the values of the other parameters such as the drift rate variability (*Var*(*v*)) or an overall reduction in decision threshold (*a*) that stays constant within a trial. Figure 11 shows the results of these simulations by plotting accuracies as a function of corresponding RTs (using a bin size of 0.05 s).

Conditional accuracy curves based on simulated data showed a steadily declining accuracy with increasing RT (Figure 11). Moreover, although the rate of this decline is higher for a lower threshold parameter, a similarly increasing rate of decline is observed for higher levels of the drift rate variability parameter as well, with no modification of the threshold or any other parameter within a trial. Additionally, increasing or decreasing the starting point variability had no discriminable effect on the rate of decline in accuracy with time. These results suggest that, importantly, decreasing the constant decision threshold (i.e., without the need for within trial modulation) or increasing the variability in drift rate could underlie decreasing accuracy toward a deadline.

## Discussion

Many studies using 2AFC tasks have focused on the optimality of decisions in free response paradigms (e.g., Bogacz et al., 2006, 2010; Simen et al., 2009; Starns and Ratcliff, 2010; Balcı et al., 2011b). Some of these studies showed that with enough training human participants can optimize the speed-accuracy tradeoff in their decisions by adopting RR-maximizing decision thresholds. When response deadlines are imposed in these tasks, reward maximization instead requires the decision-maker to collapse decision thresholds within a trial such that at the time of deadline, they meet at the starting point of the evidence accumulation process. This is an adaptive process as it secures at least a 50% chance that the reward will be obtained instead of earning nothing if the decision-maker is late. Frazier and Yu (2008) showed the relevance of timing uncertainty to the parameterization of this adaptive within-trial threshold crossing process. Participants with higher timing uncertainty should start collapsing decision thresholds earlier to maximize reward. Thus, reward maximization in these tasks entails factoring timing uncertainty into decisions in a normative fashion.

To this end, previous research has shown that humans and non-human animals are able to take normative account of their endogenous timing uncertainties in both temporal and non-temporal decision making tasks (for review see Balcı et al., 2011a). This prediction was tested in the current study by examining conditional accuracy curves and evaluating how their shape depends on deadlines and participants' endogenous timing uncertainty. Although our results showed that accuracy decreased with time toward the deadline for many participants, this rate of decline was much lower than expected from an optimal decision-maker and did not correlate with measured levels of timing uncertainty. In contrast to optimal performance predictions, the timing of the onset of decline in accuracy increased rather than decreased with higher levels of timing uncertainty in the short deadline condition, when this uncertainty was quantified using a static visual stimulus, and also in the long deadline condition when it was quantified using a dynamic visual stimulus. It is possible that our analytical approach, i.e., using linear fits to accuracy levels of binned RT data, was not sensitive enough to capture such relations and might be vulnerable to artifacts depending on the number of data points included per bin. However, this relation did not hold even when the onset of this decline in accuracy was characterized by the location of peak accuracy levels using a non-binning approach. Overall, these results suggest that there is no relation between decreasing accuracy and timing uncertainty. Importantly, however, our analyses showed that slopes were less negative in the long deadline condition compared to the short deadline condition, suggesting that interval timing still had an effect on participants' choice behavior.

There are at least three possible explanations for sub-optimal behavior in the deadline blocks. First, participants may have kept favoring accuracy over reward rate throughout the experiment, which has been previously reported (e.g., Maddox and Bohil, 2004; Bogacz et al., 2006, 2010; Balcı et al., 2011b). Thus, accuracy bias could have prevented within trial modulation of thresholds to reduce overall error rates. This possibility relies on the implicit assumption that errors are subjectively more costly than missed trials. Second, participants may have started collapsing thresholds later than the optimal case due to underestimation of the deadline. In this case, accuracy would remain above the chance level at the time of response deadline. However, our analyses did not support this possibility. Third, sub-optimal decision making may be caused by mechanistic limitations at the neuronal level which may not allow for within-trial decision threshold modulation, at least for decisions made in less than one second. This is a plausible explanation of our results, given that the cognitive cost (i.e., executive load) of modulating the value of the decision threshold in real-time may outweigh its benefits in terms of increasing the overall reward attained throughout a session. Importantly, participants differed in terms of decreasing and increasing accuracy with time (see Figure 9, where some participants' accuracies increased rather than decreased toward the deadline), which could again be explained by individual differences in bias toward accuracy, as opposed to maximizing reward.

Slower RTs on error trials are commonly found in 2AFC research with free responding (Ratcliff and Rouder, 1998; Ratcliff and McKoon, 2008). These patterns can be accounted for by the extended DDM by allowing the drift rate to vary between trials. Drift variability enables the extended DDM to account for slower average error RTs than correct RTs. Inflation of this variability parameter (in addition to decreasing the constant threshold) should therefore produce decreasing accuracy with slower RTs in conditional accuracy curves, even in the absence of collapsing thresholds within a trial. Our simulations confirmed that accuracy can decline steadily with RT without any accompanying threshold collapse. We have shown that, while a concomitantly decreasing threshold parameter yields an additionally higher rate of decline in accuracy, a similar effect is observed by increasing drift rate variability across trials, whereas modifying starting point variability had no such effect. This lack of a visible effect of the starting point parameter on the rate of decline in accuracy with time was expected, given that increasing this parameter results in faster error RTs, which should not necessarily translate into slower error RTs when the same parameter is decreased. Overall, these results suggest that increasing drift rate variability or setting the constant decision threshold to a lower value might be a way to mimic the effect of collapsing thresholds on accuracy without actually collapsing them.

Finally, it is also important to note that a cross-over between faster and slower error responses has been suggested depending on the difficulty of the task (see Luce, 1986). Namely, harder tasks (i.e., higher error rates) have been shown to lead to slower RTs for error trials, whereas participants had faster error RTs in easier tasks (e.g., Ratcliff and Rouder, 1998). It is possible that our task was a relatively easy one, given the low error rates observed (Figure 6), the small number of trials in the last RT bin of the conditional accuracy curves (Figure 9), and a relatively high estimated drift rate (i.e., 0.4447) (see Section Drift-Diffusion Model Simulations). However, we still observe slower RTs for error trials, as can be seen in Figure 8. Therefore, studies using an easier task still may not observe a more pronounced decline in accuracy with time, but this remains an open question.

Future studies should increase the cost of missing a deadline by explicitly adding a penalty. Under such payoff structures, one might be more likely to observe threshold collapsing. However, note that in these cases the optimal threshold collapse trajectories will also change (possibly meeting prior to the response deadline) due to the explicit penalty for late responses. Additionally, speed-accuracy tradeoff functions in tasks that use response signal methodology do not exhibit reduction in accuracy with increasing lags (e.g., Wickelgren, 1977). On the other hand, in our free response paradigm, such decline in accuracy was apparent in conditional accuracy curves. Response signal paradigms typically employ a single signal (or a series of equally distributed signals) after which the participant is instructed to respond as soon as possible, ensuring that there are no fast guesses, in addition to making within trial strategic manipulation of decision making parameters harder (Heitz, 2014). This difficulty is due to the fact that, by the time the response signal is given, subjects need to make a choice using the already accumulated (and potentially partial) evidence. This approach contrasts with the one we have used in a number of ways. First, subjects do not necessarily need to keep track of the time to respond in response signal tasks, whereas in our experimental design, participants needed to constantly rely on endogenous markers of the passage of time in order to maximize reward, which is likely more taxing in terms of information processing throughout the decision process. In turn, the relatively higher amount of cognitive resources available to the decision maker in the response signal paradigm might present itself as lower variability in drift rate, which as we showed can underlie declining accuracy with time. Secondly, the response signal paradigm allows post-signal accumulation of evidence to a certain extent, whereas our methodology does not permit it at all. As a result, one might expect that, even if participants were able to modulate thresholds within a trial (which we show here to not be the case), giving the chance to accumulate more evidence after a response signal might obscure a decline in accuracy with slower RTs. Further empirical work is needed to elucidate the possible sources of these differences between the two experimental paradigms, although the similarity of the implementation of SAT by decision makers has been questioned due to fundamental differences in the two approaches (see Heitz, 2014).

Overall, our empirical results do not support the optimal performance predictions regarding within-trial collapsing of thresholds under response deadlines. A slight decline in accuracy was observed for decisions made near the response deadlines; however, this decline never reached chance level, which is predicted by optimal threshold collapse. Moreover, the observed decline in accuracy was not related to the level of endogenous timing uncertainty in the expected direction, and it could be accounted for by DDM parameters that are constant within trials.

## Author note

A version of the abstract of this paper was previously published in: Karşılar, H., Simen, P., Papadakis, S. and Balcı, F. (2014). Procedia - Social and Behavioral Sciences, 126, 201

### Conflict of interest statement

The authors declare that the research was conducted in the absence of any commercial or financial relationships that could be construed as a potential conflict of interest.
