# Supplementary material for: Speed accuracy trade-off under response deadlines
Source: Front Neurosci. 2014 Aug 15;8:248. doi: 10.3389/fnins.2014.00248 (PMC4133757; doi:10.3389/fnins.2014.00248)
Supplement: Supplementary file 1 [file DataSheet1.PDF]

Supplementary Material

Speed accuracy trade-off under response deadlines

Hakan Karşılar[1], Patrick Simen[2], Samantha Papadakis[2], Fuat Balcı[1]*,

[1]Koç University, Department of Psychology, Istanbul, Turkey
[2]Oberlin College, Department of Neuroscience, Oberlin, OH, USA

*** Correspondence:** Fuat Balcı, Koç University, Department of Psychology, Rumelifeneri Yolu, Sariyer, Istanbul, 34450, Turkey.
fbalci@ku.edu.tr

1. Supplementary Data

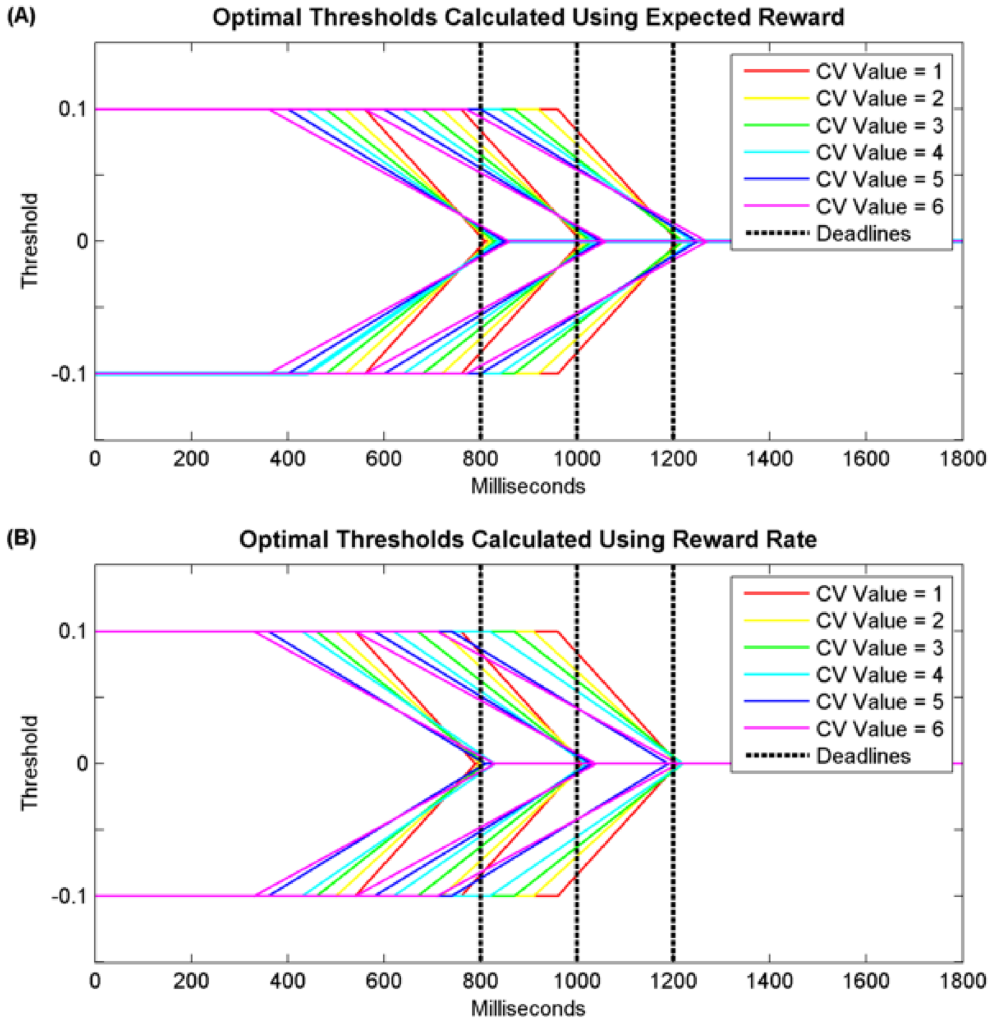

Supplementary Figure 1: Optimal threshold collapse trajectories selected from the family of linear decline functions for three different response deadlines and six hypothetical levels of timing uncertainty when optimality criterion is taken as (A) the expected total reward, and (B) reward rate. Vertical dashed lines represent the response deadlines (800, 1000 & 1200 ms).

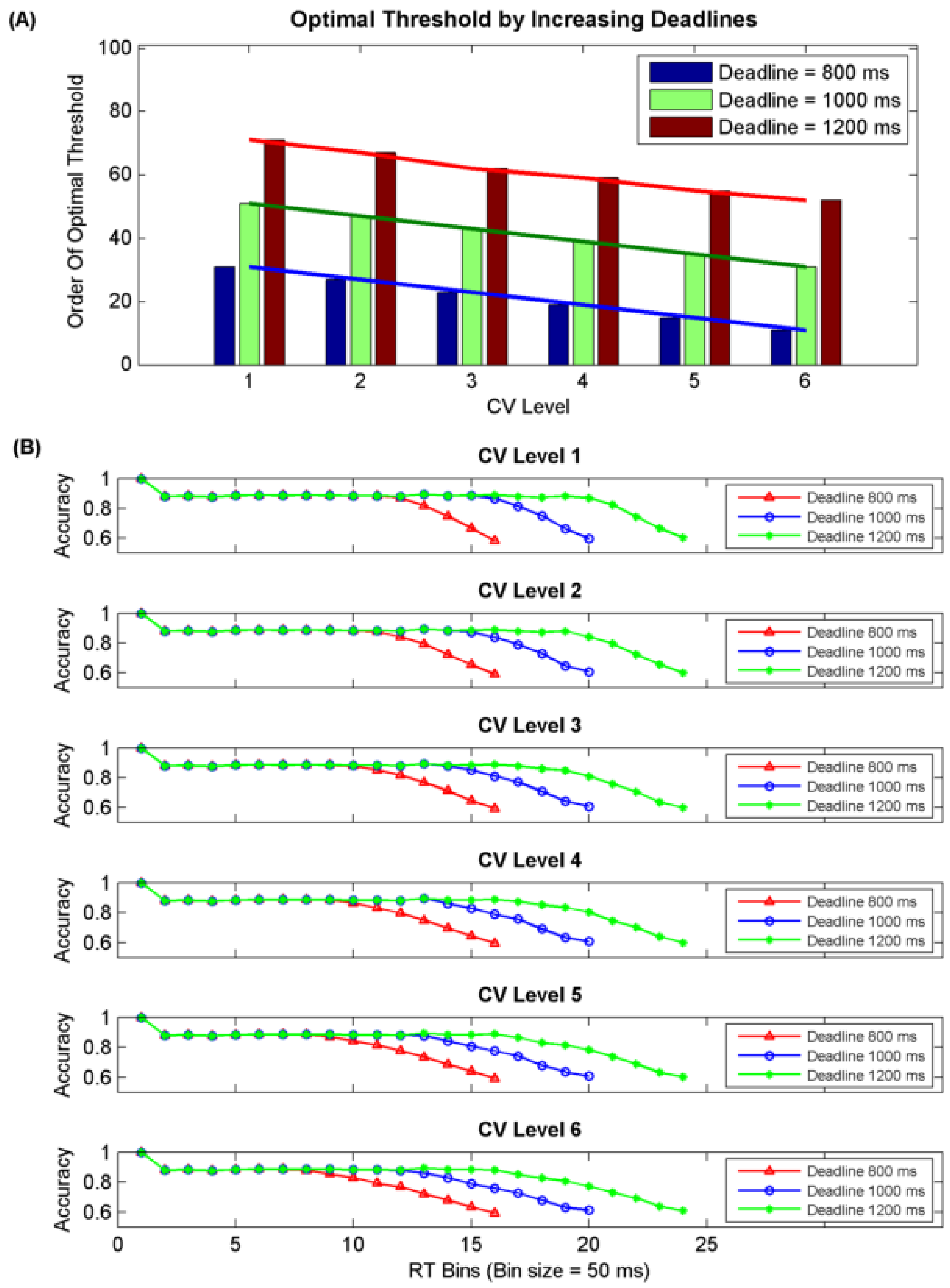

Supplementary Figure 2: (A) Bar graphs depicting the order of the optimal threshold collapse trajectories (out of 101 thresholds with 0.01 s increments) selected from the family of linear decline

functions for six hypothetical levels of timing uncertainty. Lines connect the bars. (B) Conditional accuracy curves for the six CV conditions, shown separately for the three response deadlines. Red lines represent the conditional accuracy curves for the short deadline (800 ms), blue lines for the medium deadline (1000 ms), and green lines for the long deadline (1200 ms). (Both figures 2A & 2B are based on expected total reward as the optimality criterion.)

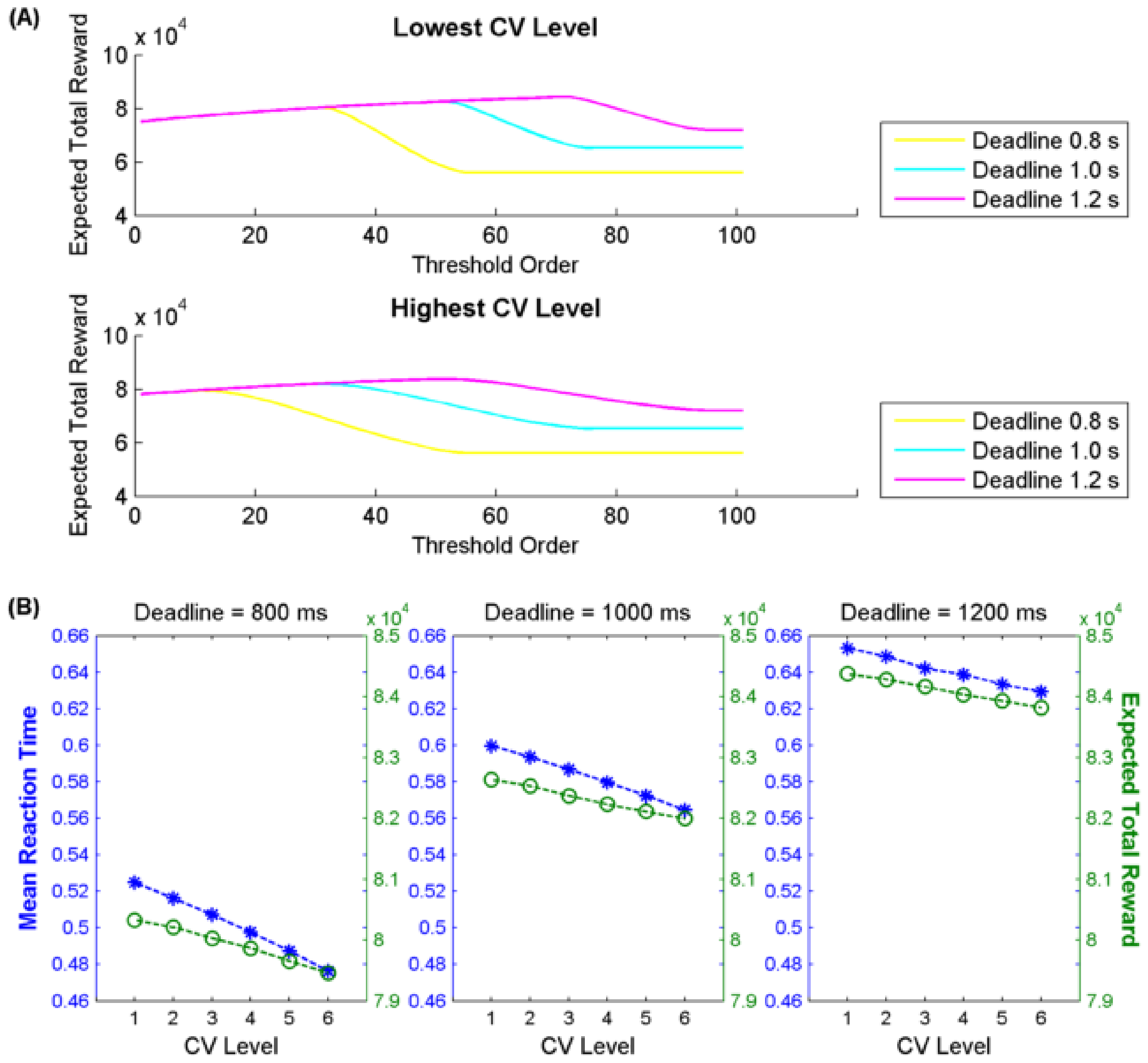

Supplementary Figure 3: (A) Expected total reward amount for the highest and lowest CV levels as a function of the order of threshold among the 101 thresholds tested (here defined as "Threshold Order"). (B) Mean reaction times and expected total reward amounts as a function of six levels of CV defining six linear threshold collapse trajectories for the short (800 ms), medium (1000 ms) and long (1200) simulated deadlines. (Both figures 3A & 3B are based on expected total reward as the optimality criterion.)
